# Anisotropy, Anatomical Region, and Additional Variables Influence Young's Modulus of Bone: A Systematic Review and Meta‐Analysis

**DOI:** 10.1002/jbm4.10835

**Published:** 2023-10-31

**Authors:** Krisztián Kovács, Szilárd Váncsa, Gergely Agócs, Andrea Harnos, Péter Hegyi, Viktor Weninger, Katinka Baross, Bence Kovács, Gergely Soós, György Kocsis

**Affiliations:** ^1^ Department of Orthopaedics Semmelweis University Budapest Hungary; ^2^ Centre for Translational Medicine Semmelweis University Budapest Hungary; ^3^ Institute for Translational Medicine, Szentágothai Research Centre, Medical School University of Pécs Pécs Hungary; ^4^ Division of Pancreatic Diseases, Heart and Vascular Center Semmelweis University Budapest Hungary; ^5^ Department of Biophysics and Radiation Biology Semmelweis University Budapest Hungary; ^6^ Department of Biostatistics University of Veterinary Medicine Budapest Hungary

**Keywords:** BONE ELASTICITY, BONE MODELING, FINITE ELEMENT ANALYSIS, IMPLANT RESEARCH, YOUNG'S MODULUS

## Abstract

The importance of finite element analysis (FEA) is growing in orthopedic research, especially in implant design. However, Young's modulus (*E*) values, one of the most fundamental parameters, can range across a wide scale. Therefore, our study aimed to identify factors influencing *E* values in human bone specimens. We report our systematic review and meta‐analysis based on the recommendation of the Preferred Reporting Items for Systematic Reviews and Meta‐Analyses (PRISMA) 2020 guideline. We conducted the analysis on November 21, 2021. We included studies investigating healthy human bone specimens and reported on *E* values regarding demographic data, specimen characteristics, and measurement specifics. In addition, we included study types reporting individual specimen measurements. From the acquired data, we created a cohort in which we performed an exploratory data analysis that included the explanatory variables selected by random forest and regression trees methods, and the comparison of groups using independent samples Welch's *t* test. A total of 756 entries were included from 48 articles. Eleven different bones of the human body were included in these articles. The range of *E* values is between 0.008 and 33.7 GPa. The *E* values were most heavily influenced by the cortical or cancellous type of bone tested. Measuring method (compression, tension, bending, and nanoindentation), the anatomical region within a bone, the position of the bone within the skeleton, and the bone specimen size had a decreasing impact on the *E* values. Bone anisotropy, specimen condition, patient age, and sex were selected as important variables considering the value of *E*. On the basis of our results, *E* values of a bone change with bone characteristics, measurement techniques, and demographic variables. Therefore, the evaluation of FEA should be performed after the standardization of in vitro measurement protocol. © 2023 The Authors. *JBMR Plus* published by Wiley Periodicals LLC on behalf of American Society for Bone and Mineral Research.

## Introduction

The rate of orthopedic prosthetic implantations is increasing in parallel with the number of arthritic joints in elders. This increase will be more than 670% and 170% in hip and knee arthroplasties by 2030, respectively. Therefore, the rising implantations and the developing technology open new opportunities for custom‐made implants and new implant research.^[^ ^1^ ^]^

One method that helps research new implant designs is finite element analysis (FEA). FEA is a numerical stress analysis technique that allows injury analysis, and presurgery simulation to model the behavior of implant fitting in bone.^[^ ^2^, ^3^, ^4^ ^]^ During FEA, the examined region is divided into elements, with the proper material properties of each.^[^ ^5^ ^]^ To recreate the appropriate virtual model, it is crucial to have precise material properties for each element. However, the bone is an anisotropic material; therefore, the elasticity of bone (expressed with Young's modulus; *E* value, which measures the tensile or compressive stiffness of a solid material when force is applied lengthwise) depends on the measuring orientation.^[^ ^6^, ^7^, ^8^ ^]^ Ignoring anisotropy causes up to 50% of relative error by FEA.^[^ ^4^ ^]^ Furthermore, the elasticity of bone can be measured by several in vitro or in vivo methods. The computed tomography (CT)‐based estimation of Young's modulus has limited value, due to the inability of capturing non‐homogeneous microstructures, as the bone is an anisotropic material.^[^ ^9^ ^]^ On the other hand, the most common in vitro bone specimen measurements are micro–macro mechanical, ultrasonic (US), and nanoindentation tests.^[^ ^9^, ^10^ ^]^

The reliability of these models depends, among other factors, on the accuracy of the input parameters. However, *E* values vary in a wide range in the literature. On the basis of the study by Wu et al.,^[^ ^10^ ^]^ *E* values ranged between 1.28 and 30.6 GPa in trabecular bones in the same anatomical region. In another review by Nobahkti et al.,^[^ ^11^ ^]^ a 0.61–25 GPa range of *E* values was reported in bulk scale specimens, but some other studies reported 60 GPa values in their results.^[^ ^11^, ^12^, ^13^ ^]^

In light of these contradictory results, we aimed to investigate factors influencing *E* values in human bones. We hypothesized that the *E* value of a bone correlated with demographic data, specimen characteristics, and measurement specifics.

## Methods

We performed our systematic review and meta‐analysis based on the recommendation of the Preferred Reporting Items for Systematic Reviews and Meta‐Analyses (PRISMA) 2020 guideline. (Table S1),^[^ ^14^ ^]^ whereas we followed the Cochrane Handbook.^[^ ^15^ ^]^ Furthermore, we registered the study protocol on PROSPERO (registration number CRD42021286292). We included only case series and case reports in our analysis reporting on individual measurements.

### Information sources and search strategy

We conducted our systematic search on November 21, 2021. The used databases were PubMed, Central Library (Cochrane), and Embase. During the systematic search, we used the following search key: “bone AND (trabecula* OR cortical OR trabeculae OR cancellous) AND (elastic* OR young OR modulus OR “mechanical properties”). No publication date, language, or any other filters were applied during the search.

### Selection process

We used the Endnote version 21.0 (Clarivate Analytics, Philadelphia, PA, USA) reference manager software for the selection. After the removal of duplicate references (manual and automatic), the selection was performed by two independent review authors (KK and VW) by reference title and abstract. At this point, Cohen's kappa coefficient for the selection was 0.99. Next, the same authors made the full‐text selection with a 0.84 Cohen's kappa coefficient value. A third review author resolved disagreements.

### Data collection process and data items

From the eligible articles, data were collected by four authors (KK, KB, BK, GS) in pairs. Each entry was extracted by one review author and verified by another. Ambiguous data were reviewed by all authors to minimize the risk of extraction error.

We extracted the following data: first author, country, the year of publication, repetition count in case of repeated measurements, specimen size (each dimension), exact anatomical location, sex, age, measuring method and equipment type, the condition of the specimen, the direction of loading (with respect to the in situ anatomical orientation: anteroposterior [AP], axial, mediolateral [ML]) and, finally, the *E* values recorded in GPa. Each extracted entry corresponds either to a single measurement or to the mean of a series of repeated measurements carried out on a specimen. A specimen is a portion of a bone cut and prepared for measurement.

### Eligibility criteria

Eligible articles included healthy human bones reporting *E* values based on different variables. In order to reach our goal, we included articles that contained human bone elastic modulus results from non‐imaging techniques, such as in vitro testing machines. Articles with animal specimens, imaging based measurement techniques, and patients who had any kind of hormonal, corticosteroid, or other bone‐affecting therapy were excluded. Patients with bone tumors or any bone disease were also excluded together with pediatric patients (i.e., males below 16 years and females below 14 years).^[^ ^16^ ^]^ We included case reports, case series, and cohort analyses with individual patient data, regarding demographic data, bone type, specimen size and condition, measuring method, and loading orientation. Conference abstracts or studies with unavailable full text were excluded.

### Study risk of bias assessment

Four authors (KK, KB, BK, GS) independently performed the risk of bias assessment. Each article was assessed in duplicate using the Case Reports (CARE) tool.^[^ ^17^ ^]^ Domain numbers 7 and 10 were omitted (details in Data S1). Disagreements were resolved by consensus discussion with a third experienced reviewer.

### Synthesis methods

For primary data extraction and organization Microsoft Office Excel (Microsoft Office Professional Plus 2013; Microsoft, Redmond, WA, USA) was used. Statistical analysis was carried out in Excel and R (R Core Team 2022, v4.1.3; R Foundation for Statistical Computing, Vienna, Austria; https://www.r-project.org/). To fit a random forest and calculate variable importance, the functions of the “party” package (version 1.3‐10) were used.^[^ ^18^, ^19^, ^20^ ^]^ There was an attempt to build a statistical model describing the dependence of the magnitude of *E* value on different explanatory variables; however, the structure of the extracted data did not enable this (Table S4). Instead, we carried out a series of systematic pairwise comparisons with various subgroups of the explanatory variable values, we reported mean ± standard deviation, and the significance of the difference between the means of the subgroups was tested by Welch's *t* test with a level of significance of 0.05. During the analysis, a 10% difference was considered as clinically relevant in *E* values between the analyzed groups. Additionally, anisotropy was investigated by the Bland–Altman method where data differing only in loading orientation were available for the same specimen.^[^ ^21^ ^]^ Finally, the variable importance measure was calculated for the explanatory variables.^[^ ^19^, ^20^, ^22^ ^]^

## Results

### Search and selection

**Fig. 1:**
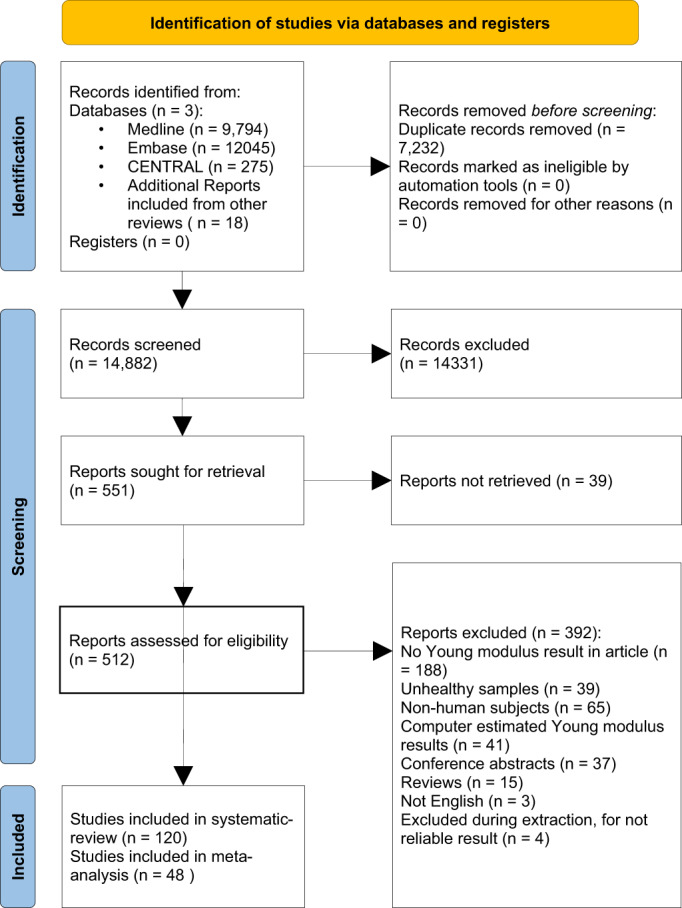
Preferred Reporting Items for Systematic Reviews and Meta‐Analyses (PRISMA) 2020 flowchart representing the study selection process.

Altogether 22,114 records were identified using our search key, and finally, 48 articles contained individual results of *E* values (Fig. 1).^[^ ^23^, ^24^, ^25^, ^26^, ^27^, ^28^, ^29^, ^30^, ^31^, ^32^, ^33^, ^34^, ^35^, ^36^, ^37^, ^38^, ^39^, ^40^, ^41^, ^42^, ^43^, ^44^, ^45^, ^46^, ^47^, ^48^, ^49^, ^50^, ^51^, ^52^, ^53^, ^54^, ^55^, ^56^, ^57^, ^58^, ^59^, ^60^, ^61^, ^62^, ^63^, ^64^, ^65^, ^66^, ^67^, ^68^, ^69^, ^70^ ^]^

### Characteristics of the included studies

The main characteristics of these articles are summarized in Table 1. The years of publications ranged from 1966 to 2020. The articles were published in 13 countries, the United States (*n* = 20), France (*n* = 7), and Belgium (*n* = 3) being the most frequent ones.

**Table 1:** Main characteristics of the included studies

Author	Country	Element number	Specimen size	Bone specimen origin	Sex (male, female, mixed, NA)	Age (mean, years)	Testing method	Specimen condition	Orientation of loading
Anglin et al.^[^ ^23^ ^]^	Canada	10	1 mm < <5 mm	Scapula	Female	80	Nanoindentation	Dry	Side
Ashman et al.^[^ ^24^ ^]^	USA	3	1 mm < <5 mm	Femur	Na	NA	UH	Wet	Non defined
Banse et al.^[^ ^25^ ^]^	Belgium	20	>5 mm	Femur	Male	63	Compression	Wet	Non defined
Bargren et al.^[^ ^26^ ^]^	UK	2	1 mm < <5 mm	Femur	Female	23	Tension	Wet, dry	Axial
Bensamoun et al.^[^ ^27^ ^]^	France	1	1 mm < <5 mm	Femur	Male	70	UH	Wet	Axial
Bensamoun et al.^[^ ^28^ ^]^	France	3	1 mm < <5 mm	Femur	Male	70	Nanoindentation	Wet, dry	Axial
Berteau et al.^[^ ^29^ ^]^	France	34	1 mm < <5 mm	Fibula	Mixed	40	UH, 3p‐bending	Wet	Side, non‐defined
Bini et al.^[^ ^30^ ^]^	Italy	3	<1 mm	Femur	NA	NA	Pressure, tension	NA	Non defined
Birnbaum et al.^[^ ^31^ ^]^	Germany	21	>5 mm	Femur	NA	73	Compression	NA	Non defined
Bry et al.^[^ ^32^ ^]^	France	12	1 mm < <5 mm	Humerus	Male	65	Compression, tension	NA	AP, axial
Carretta et al.^[^ ^33^ ^]^	Switzerland	1	<1 mm	Femur	Female	56	Tension, 3p‐bending	Dry	Non defined
Choi et al.^[^ ^34^ ^]^	USA	2	<1 mm	Tibia	Male	60	3p‐bending	Wet	Non defined
Cuppone et al.^[^ ^35^ ^]^	UK	30	1 mm < <5 mm	Femur	Mixed	78	3p‐bending	Wet	Non defined
Dall'ara et al.^[^ ^36^ ^]^	Austria	12	1 mm < <5 mm	Vertebra	Mixed	80	Nanoindentation	Dry	AP, side, axial
Dong et al.^[^ ^37^ ^]^	USA	18	1 mm < <5 mm	Femur	NA	56	Tension	NA	AP
Ducheyne et al.^[^ ^38^ ^]^	Belgium	15	1 mm < <5 mm	Femur	Male	47	Compression	Wet	Axial
Dunham et al.^[^ ^39^ ^]^	Canada	7	1 mm < <5 mm	Humerus	Female	79	Compression	Wet	Axial
Evans et al.^[^ ^40^ ^]^	USA	6	1 mm < <5 mm	Fibula	Male	57	Tension	Wet	Non defined
Fan et al.^[^ ^41^ ^]^	USA	1	<1 mm	Tibia	Male	52	Nanoindentation	Dry	Non defined
Fan et al.^[^ ^42^ ^]^	USA	2	1 mm < <5 mm	Tibia	Male	52	Nanoindentation	Dry	AP, side, axial
Franzoso et al.^[^ ^43^ ^]^	Italy	1	1 mm < <5 mm	Femur	Male	81	Nanoindentation	Dry	Side, axial
Guérard et al.^[^ ^44^ ^]^	France	12	>5 mm	Calcaneus	Mixed	81	Compression	Wet	Non defined
Hengsberger et al.^[^ ^45^ ^]^	Switzerland	2	1 mm < <5 mm	Femur	Female	86	Nanoindentation	Dry	Axial
Jensen et al.^[^ ^46^ ^]^	Denmark	4	>5 mm	Tibia	NA	68	Compression	Wet	Axial
Katsamanis et al.^[^ ^47^ ^]^	USA	1	>5 mm	Femur	Male	50	Tension	Dry	Axial
Keller et al.^[^ ^48^ ^]^	USA	9	1 mm < <5 mm	Femur	Mixed	62	4p‐bending	Wet	AP
Korsa et al.^[^ ^49^ ^]^	Czech Republic	3	<1 mm	Humerus	Male	37	Nanoindentation	NA	AP, side, axial
Kuhn et al.^[^ ^50^ ^]^	USA	5	<1 mm	Iliac crest, femur	Male	39	3p‐bending	Wet	Non defined
Mansat et al.^[^ ^51^ ^]^	France	6	>5 mm	Scapula	NA	NA	Uh	Wet	AP, side, axial
Martens et al.^[^ ^52^ ^]^	Belgium	45	>5 mm	Femur	Mixed	55	Compression	Wet	AP, side, axial
Moreschi et al.^[^ ^53^ ^]^	France	3	NA	Calcaneus	NA	79–88	Compression	Wet	Axial
Nomura et al.^[^ ^54^ ^]^	Japan	12	>5 mm	Mandibula	Female	66	Uh	Wet	Non defined
Odgaard et al.^[^ ^55^ ^]^	USA	17	1 mm < <5 mm	Tibia	Male	54	Compression	Wet	Non defined
Odgaard et al.^[^ ^56^ ^]^	Denmark	18	>5 mm	Tibia	Male	55	Compression	Wet	Axial
Pattijn et al.^[^ ^57^ ^]^	Belgium	1	NA	Femur	NA	NA	Compression, UH	Wet	Non defined
Pattin et al.^[^ ^58^ ^]^	USA	9	1 mm < <5 mm	Femur	Male	31	Compression, strain	Wet	Axial
Reilly et al.^[^ ^59^ ^]^	USA	19	1 mm < <5 mm	Femur	NA	53	Strain/compression	Wet	Axial
Reilly et al.^[^ ^60^ ^]^	USA	11	1 mm < <5 mm	Femur	Mixed	21–71	Strain/compression	Wet	Axial
Reisinger et al.^[^ ^61^ ^]^	Austria	1	1 mm < <5 mm	Femur	Female	61	Strain	Wet	Non defined
Rho et al.^[^ ^62^ ^]^	USA	9	1 mm < <5 mm	Femur	Male	55	Nanoindentation	Dry	Non defined
Rho et al.^[^ ^63^ ^]^	USA	2	<1 mm	Tibia	NA	60	Strain, UH	Wet	Non defined
Rho et al.^[^ ^64^ ^]^	USA	4	1 mm < <5 mm	Tibia, vertebra T12	Male	59	Nanoindentation	Dry	Axial, non‐defined
Rho et al.^[^ ^65^ ^]^	USA	1	<1 mm	Femur	Male	56	Nanoindentation	Wet	Axial
Sedlin et al.^[^ ^66^ ^]^	Sweden	14	1 mm < <5 mm	Femur	Mixed	59	3p‐bending	Wet	Non defined
Stern et al.^[^ ^67^ ^]^	USA	10	1 mm < <5 mm	Femur	Male	49	Strain	Wet	Non defined
Subit et al.^[^ ^68^ ^]^	USA	6	<1 mm	Rib VI‐VII	NA	64	Strain	Wet	Non defined
Winwood et al.^[^ ^69^ ^]^	UK	2	1 mm < <5 mm	Femur	Female	54	Strain, compression	Wet	Axial
Zysset et al.^[^ ^70^ ^]^	USA	8	<1 mm	Femur	Mixed	75	Nanoindentation	Wet	Non defined

NA, not applicable.

### Characteristics of the cohort

Detailed article characteristics are shown in Table 2. We had bone measurements in a total of 756 entries from 397 specimens. The most frequent measurement techniques were compressional, nanoindentation, and tensional tests. The majority of specimens were cortical, and wet, and the three most commonly tested bones were the femur, tibia, and fibula. The range of age was between 15 and 96 years. More than half of the specimens came from male subjects, and the range of specimens was between 0.035 and 20 mm.

**Table 2:** Detailed characteristics of the acquired cohort

		*n*	Mean E (Gpa)	SD (Gpa)	Min (Gpa)	Max (Gpa)
All bone	Distinct donor	212				
	Specimen	397	‐	‐	‐	‐
	Entry count	756	10.61	±9.22	0.008	33.70
	Femur	419	12.17	±9.29	0.008	27.00
	Tibia	125	6.46	±9.43	0.073	27.10
	Fibula	58	13.47	±4.6	3.000	21.60
	Vertebra	44	12.56	±2.61	5.320	15.72
	Humerus	40	14.99	±7.51	0.117	24.10
	Scapula	28	0.19	±0.12	0.010	0.42
	Talus	27	0.35	±0.25	0.068	1.22
	Calcaneus	15	0.24	±0.12	0.080	0.51
	Mandibula	12	24.97	±5.96	14.400	33.70
	Rib	6	13.52	±2.59	11.400	18.50
	Iliaca	4	4.05	±0.93	3.030	5.26
Sample conditions	Wet	541	8.56	±8.98	0.008	33.70
	Dry	144	17.80	±7.12	0.010	27.10
	NA	93	11.44	±7.69	0.118	24.10
Sex	Male	440	12.66	±9.23	0.008	27.10
	Female	98	9.67	±9.01	0.010	33.70
	NA	240	7.25	±8.2	0.068	21.35
Anisotropy	Axial	360	10.44	±9.75	0.019	27.10
	AP	54	9.15	±5.39	0.008	19.70
	ML	74	5.74	±5.67	0.010	18.50
	NA	290	12.35	±9.35	0.080	33.70
Sample size	<1 mm	108	15.64	±6.58	1.150	26.80
	>1 mm	665	9.87	±9.32	0.008	33.70
	NA	5	0.35	±0.17	0.148	0.54
Macrostructure	Cortical	437	16.78	±6.03	0.107	27.10
	Cancellous	341	2.70	±5.9	0.008	33.70
Age	<40 years	111	12.62	±8.4	0.071	25.00
	>60 years	375	9.47	±9.2	0.008	33.70
	Other	264	12.12	±9.23	0.019	27.10
	NA	28	2.28	±4.48	0.148	13.10
Method	Compression	302	2.96	±6.33	0.008	23.50
	Nanoindentation	203	18.22	±6.42	0.010	27.10
	Tension	114	15.44	±4.34	1.890	23.41
	Bending	88	10.75	±7.38	0.107	21.35
	Ultrasound	71	13.50	±9.3	0.148	33.70
Anatomy location	Proximal	180	2.10	±4.3	0.008	22.50
	Distal	143	7.03	±9.19	0.019	24.66
	NA	455	15.10	±7.66	0.010	33.70
Within bone	Diaphysis	392	16.53	±6.28	0.107	27.10
	Epiphysis	193	0.75	±2.14	0.010	22.50
	Metaphysis	48	3.10	±4.84	0.008	16.24
	Lumbar	30	12.19	±2.79	5.320	15.72
	Thoracolumbar	14	13.36	±2.03	8.200	15.50
	NA	101	9.21	±11.27	0.068	33.70

NA, not applicable.

### Subgroups comparisons

**Fig. 2:**
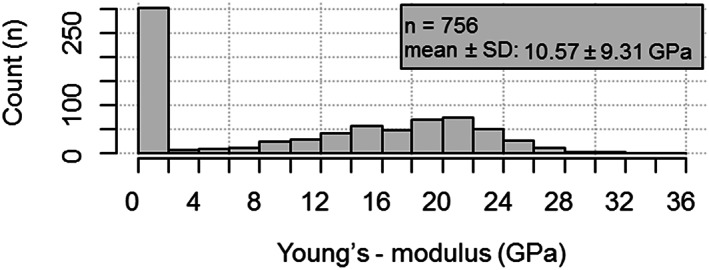
Absolute distribution of *E* values.

In the overall histogram of all entries, *E* values of cortical and cancellous specimens showed a disjunct distribution (Fig. 2). In order to achieve more homogeneous groups, we focused on the three most common bones (femur, tibia, and fibula) and investigated them by variable subgroupings or pair comparisons. The summary of the applicable findings can be seen in Table 3, and their explanatory variable comparisons are shown in Table S2.

**Table 3:** Summary of Young's modulus subgroup comparisons' applicable findings

Cortical bone tissue							
Bone region	Sample count	Age	Anisotropy	Sex	Condition	Size	Testing method
		<40 vs. >60	Axial vs. AP vs. ML	Male vs. female	Dry vs. wet	<1 mm vs. >1 mm	Bending vs. strain vs. compression vs. nanoindentation vs. US
Fibula diaphysis	58	*p* > 0.05	NA	*p* > 0.05	NA	*p >* 0.05	*p* < 0.05
Tibia epiphysis	1	NA	NA	NA	NA	NA	NA
Femur epiphysis	1	NA	NA	NA	NA	NA	NA
Femur diaphysis	274	*p* < 0.05	*p* < 0.05	*p* < 0.05	*p* < 0.05	*p* < 0.05	*p* < 0.05

Cancellous bone tissue							
Bone region	Sample count	Age	Anisotropy	Sex	Condition	Size	Testing method
		<40 vs. >60	Axial vs. AP vs. ML	Male vs. female	Dry vs. wet	<1 mm vs. >1 mm	Bending vs. strain vs. compression vs. nanoindentation vs. US
Fibula diaphysis	0	NA	NA	NA	NA	NA	NA
Tibia epiphysis	66	NA	NA	NA	NA	NA	NA
Femur epiphysis	97	NA	NA	*p* > 0.05	NA	NA	NA
Femur diaphysis	3	NA	NA	NA	NA	NA	NA

NA, not applicable.

Both femoral and fibular cortical bone specimens showed higher *E* values overall in the elder subgroup (femur: 19.35 ± 3.72 GPa for those above 60 years vs. 14.57 ± 9.21 GPa for those below 40 years; *p* < 0.001) (Table S2, rows 1–57).

For cortical bone specimens with sufficient entry counts (i.e., femur, fibula) male sex was associated with significantly higher *E* values in femoral diaphysis cortical bone (male: 19.20 ± 6.17 GPa; females: 11.25 ± 6.17 GPa; *p <* 0.001). This trend changed in the subgroup of entries measured by bending. Female specimens tended to have greater *E* values in the cortical bone of fibula and femoral cancellous bone, but without significant difference (Table S2, rows 104–159).

An appreciable number of data was only available for cortical femoral diaphysis. As for the overall comparison, the mean *E* value was higher in dry (22.42 ± 2.36 GPa) than in wet (16.87 ± 6.33 GPa) specimen entries (*p* < 0.001). This tendency was consistent for subgroup comparisons, too. (Table S2, rows 82–103).

Both in fibular and femoral (<1 mm: 20.24 ± 3.33 GPa; >1 mm: 17.22 ± 6.25 GPa; *p* < 0.001) diaphysis of cortical bones, small (<1 mm) specimen size entries had higher *E* values. However, nanoindentation measurements in femoral bone specimens showed an opposite trend, but this difference was not significant (Table S2, rows 160–194).

**Fig. 3:**
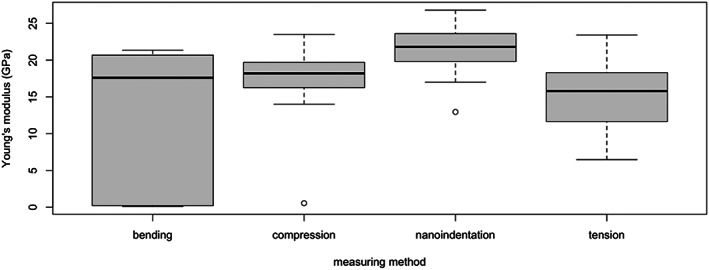
*E* values by measuring methods in cortical bone of femoral diaphysis.

A comparison was only possible for cortical specimens of the fibular and the femoral diaphysis. In femoral specimens, the nanoindentation measurements had the highest *E* values (21.71 ± 2.48 GPa), followed by compressional (17.93 ± 4.13 GPa) and tensional measurements (15.38 ± 4.24 GPa). The smallest *E* values were measured by bending (12.76 ± 8.85 GPa). US entries could not be included in the comparison due to their low number. In the fibula, bending showed smaller *E* values (9.10 ± 1.55 GPa) than United States (17.14 ± 1.89 GPa), but no other measuring method had a sufficient entry for comparisons (Table S2, rows 195–412) (Fig. 3).

**Fig. 4:**
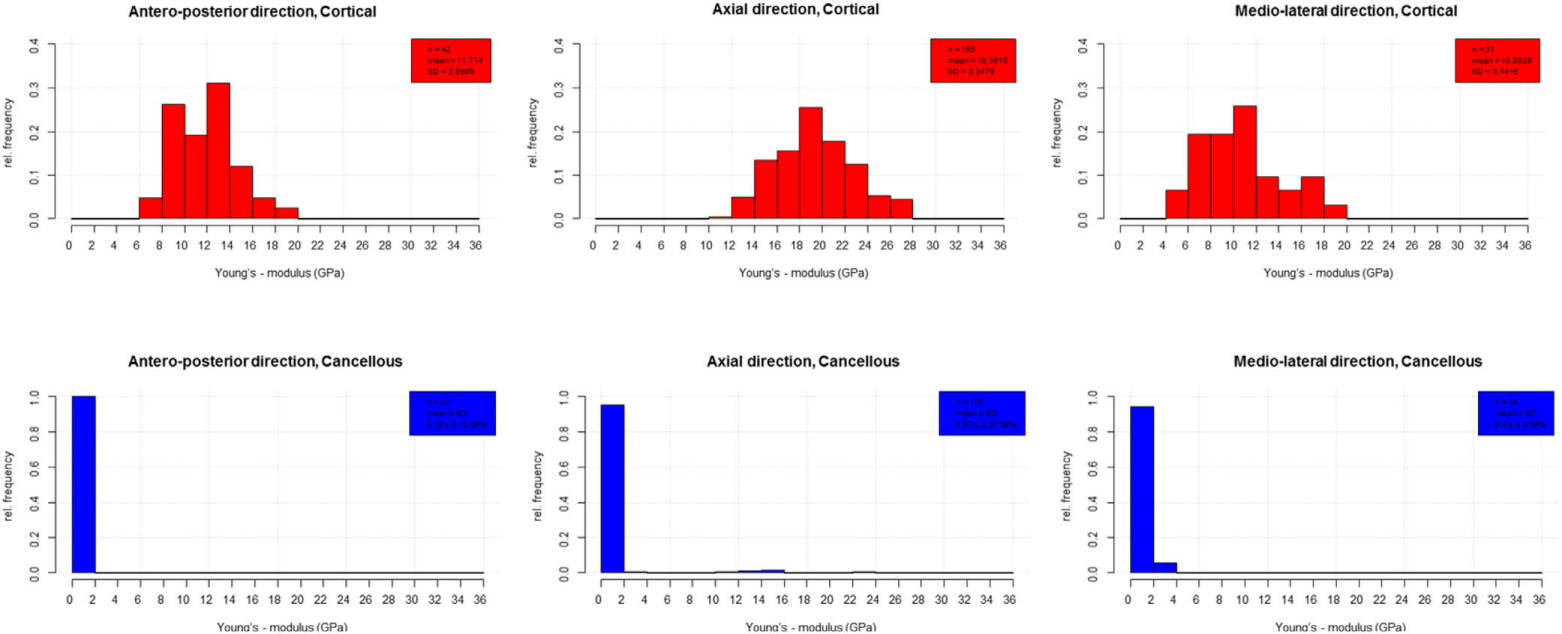
Relative distribution of cortical and cancellous bones by anisotropy.

**Fig. 5:**
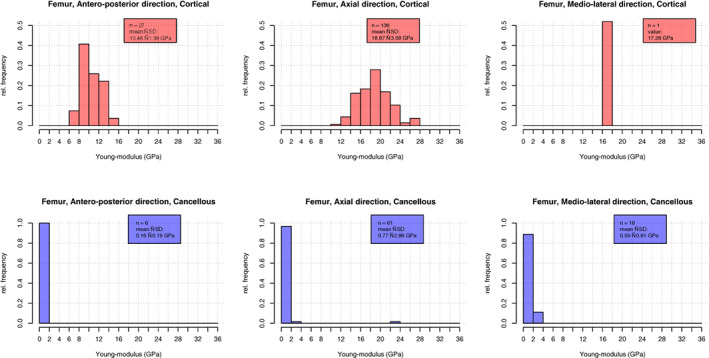
Relative distribution of cortical and cancellous femoral diaphysis by anisotropy.

**Fig. 6:**
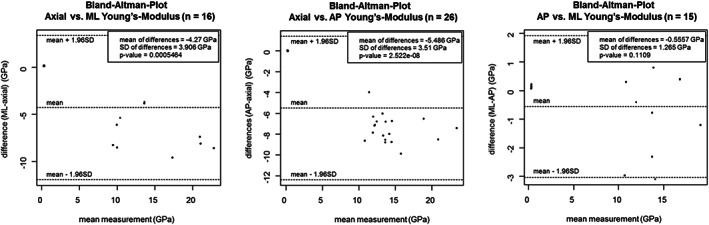
Bland–Altman‐Plots, comparing the *E* values of same specimens loaded from more than one angle.

The variation of *E* values with load orientation was investigated for all bones together as well as separately for the three most common bones. In both ways, the axially (19.39 ± 3.34 GPa) loaded specimens had the highest *E* values. Between anteroposterior (AP) (11.71 ± 2.69 GPa) and mediolateral (ML) (10.80 ± 3.44 GPa) orientations the difference was significant in cortical bones, but not in cancellous bones (Fig. 4). In case of femoral cortical bone, significant difference (*p* < 0.01) in anisotropy were seen between AP (10.46 ± 1.98 GPa) and axial (18.87 ± 3.08 GPa), and in femoral cancellous bone between axial (0.77 ± 2.86 GPa) and ML (0.59 ± 0.61 GPa) (*p* = 0.015) (Fig. 5). The figures (Fig. 6) depicting Bland–Altman plots suggested a significant difference between the mean values of axial and AP as well as of axial and ML entries. Among the most common bones, applicable data were available only in the cortical bone of femoral diaphysis for axial and AP comparisons (Table S2, rows 58–81).

Of the available data, the relationship in term of *E* value for bone regions was only possible for the cancellous bone of femoral epiphysis and metaphysis. In overall comparison, the epiphysis *E* values (0.87 ± 2.35 GPa) were three times higher (*p* = 0.010) than those of the metaphysis (3.01 ± 5.04 GPa) Nonetheless, in values by compression, the epiphysis became two times higher (0.67 ± 0.52 GPa; 0.30 ± 0.38 GPa; *p* < 0.001) (Table S2, rows 432–457).

**Fig. 7:**
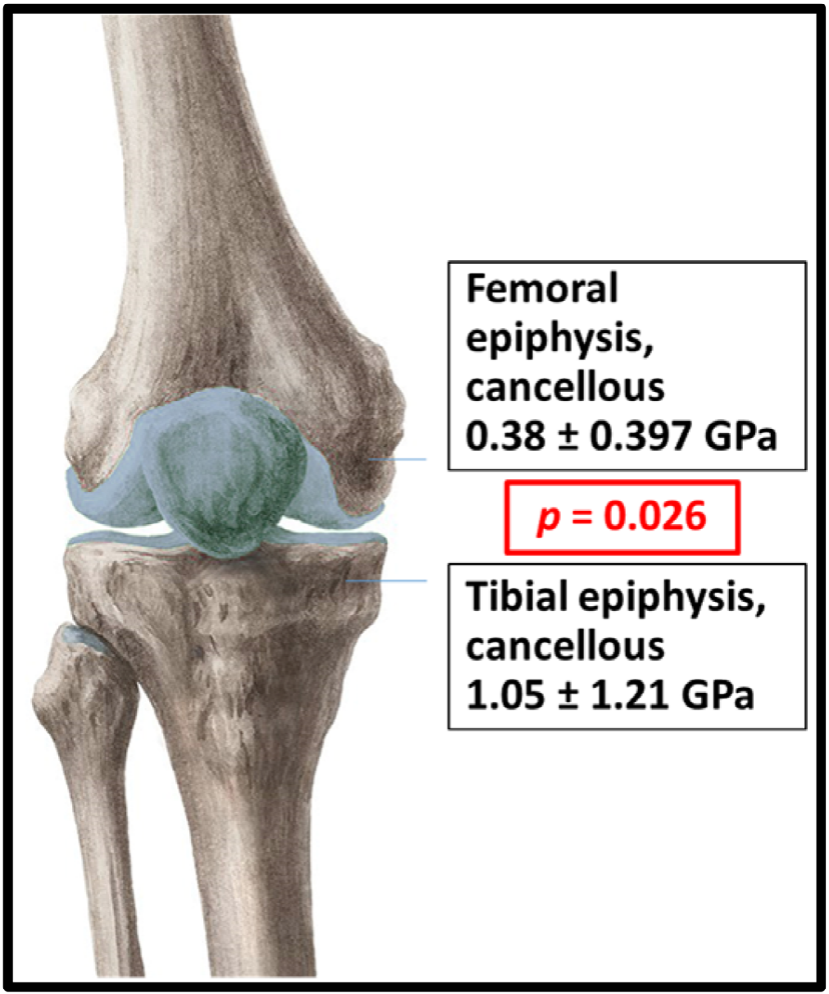
Comparison of *E* values between the femoral and tibial epiphyses.

The overall comparison of the tubular bone ends in the knee joint revealed that the epiphysis of tibia (1.05 ± 1.21 GPa) was more stiff (*p* = 0.026) than that of the femur (0.38 ± 0.39 GPa) (Table S2, rows 413–431) (Fig. 7).

### Ranking

**Fig. 8:**
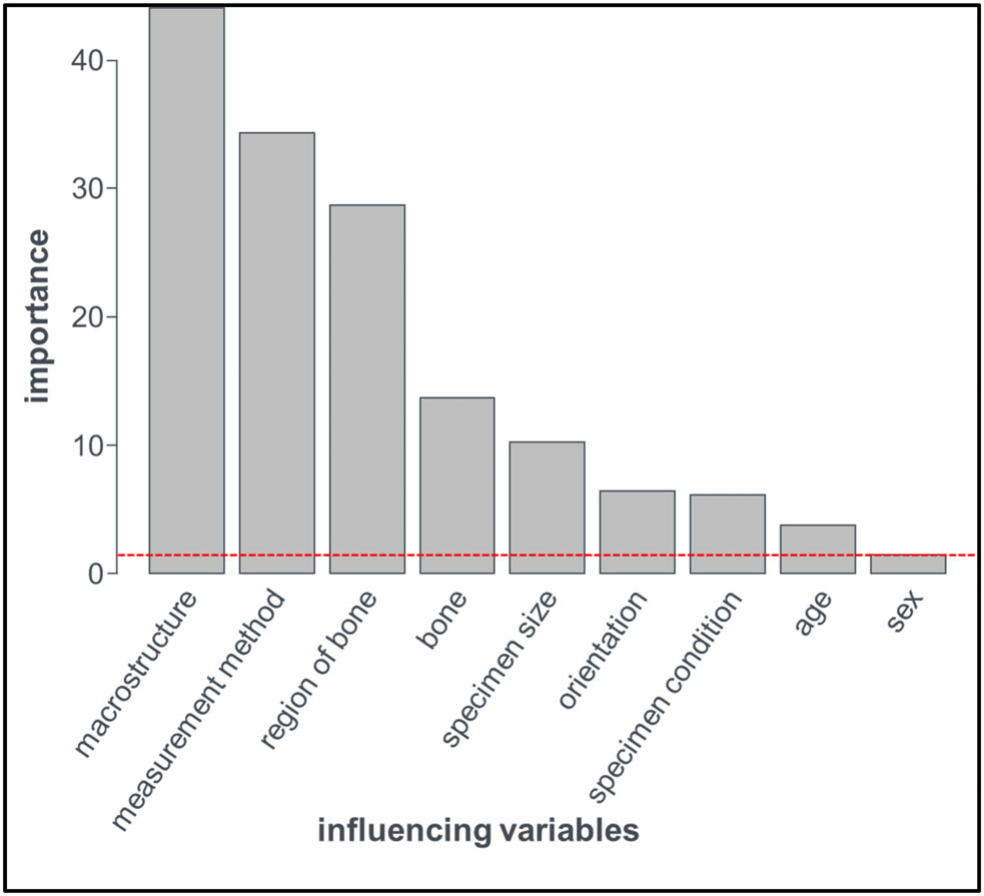
The impact of different variables on Young's modulus of bone (cutoff level marked as red broken line).

The direct regression analysis of the extracted data was not possible due to the unfavorable data structure (Table S4). However, with the help of the random forest method, the software calculated the variable importance and generated a weighted ranking of the explanatory variables of bone Young's modulus. Here, the macrostructure, the measuring method and the specific bone region had the largest impact on *E* values (Fig. 8).^[^ ^22^ ^]^

### Risk of bias assessment

The result of the risk of bias assessment is presented in Table S3 in the Supporting Information.

## Discussion

To the best of our knowledge, this is the first comprehensive literature review creating a ranking of factors influencing Young's modulus of the human bone.

In the systematic review by Helgason et al.,^[^ ^71^ ^]^ the authors investigated other methodological differences such as specimen geometry, anatomical location, and end support during testing. They faced similar limitations as we did.

Currently, no guideline can be found in the topic of standardized *E* value measurement.

The analysis of the overall data pool with random forest method demonstrated the variables with major impact on Young's modulus results of human bone specimens (Fig. 8).^[^ ^22^ ^]^ The impact of variables was in agreement with the findings of subgroup comparisons. The result that macrostructure had the largest impact was consistent with the observation by Rho et al.,^[^ ^63^ ^]^ who explained that the cortical and the trabecular bones did not behave mechanically in the same way.

Both the comparisons and the ranking method confirmed the importance of load orientation in Young's modulus. Our results were in agreement with the results of Fan et al.^[^ ^42^ ^]^ They also concluded that measurements using axial load yield higher *E* values than those using AP and ML loads. Many FEAs use isotropic and homogeneous elements^[^ ^72^, ^73^, ^74^, ^75^, ^76^ ^]^ because they make calculations faster and easier, and because the resolution of clinical quantitative computed tomography scans (QCTs) is insufficient for detecting anisotropy.^[^ ^77^ ^]^ Taddei et al.^[^ ^78^ ^]^ demonstrated that FEA results could be improved by transforming Hounsfield unit values into *E* values before sending the data for further finite element (FE) calculations. This FE model was also an isotropic model.^[^ ^78^ ^]^ Trabelsi and Yosibash^[^ ^79^ ^]^ investigated inhomogeneous and orthotropic FE models, and their conclusion was that a more realistic FEA was needed.

FE modeling is in an experimental phase for both total hip and knee prosthesis implantations.^[^ ^76^, ^80^ ^]^ However, In in silico bone models, they use isotropic and homogeneous elements.^[^ ^75^ ^]^ According to our results, however, the *E* value of the tibia in the knee joint was twice as much as that of the femur, and the anisotropic properties of the bone had to be accounted for, too.

Typically, the input data of FEA come from three‐dimensional (3D) CT. In order to create cost effective models, in vitro experiments must be correlated precisely to *E* values acquired by CT. As an example, nanoindentation measurements show significant difference to compression, bending, and tension in case of cortical specimens of femoral diaphysis (Fig. 3).

We found that wet specimens had smaller *E* values than dry ones, which was in accordance with literature.^[^ ^10^, ^45^, ^81^, ^82^ ^]^ A possible explanation for this could be the behavior of collagen. Without water, collagen fibrils might stiffen and contract axially leading to a higher bone *E* value.^[^ ^83^ ^]^

A pediatric subgroup was intended to be created; however, this was not possible due to their low number and wide age range. In the study of Ding et al.,^[^ ^84^ ^]^ a peak was observed for elders (between 40 and 50 years), and a decrease of *E* values with higher age. In our results the specimens were stiffer in the elder subgroup, but a peak like the one mentioned in the literature was not observed. Probably the fact that all the osteoporotic bone specimens were excluded caused this disagreement with other studies. In the literature, the explanations for the age‐related deterioration include the loss of trabecular bone substance,^[^ ^84^ ^]^ age‐related microdamage accumulation, and the change of collagen integrity.^[^ ^85^ ^]^ Our results contradict Nyman et al.,^[^ ^86^ ^]^ who found no variation of the *E* value with donor age in the case of nanoindentation and microindentation tests.

For our analysis we used the smallest dimension of the specimen as size parameter. According to our results, specimens with a size less than 1 mm showed higher *E* value. The reason for this was most probably the fact that small specimens were predominantly measured by nanoindentation, whereas larger specimens were tested mainly by other methods. Association with size was also found by Choi et al.,^[^ ^34^ ^]^ with a cutoff value of 0.5 mm, however, with an opposite trend: in their article the smaller specimens had smaller *E* values.

The *E* values of male and female specimens were different. A possible explanation for significantly higher values in males could be that the stiffer cortical bone may cause stress shielding. The low number of female specimens from cancellous femoral epiphysis can explain the lack of significance, but similar results were found by Vale et al.^[^ ^87^ ^]^

In the case of cortical femoral diaphysis, the true *E* value was not possible to conclude with any of the five measurement methods (the US method was not compared, due to its low entry count). Of the four different measurement methods nanoindentation tended to give the largest *E* values, whereas compression values tended to be smaller. The smallest values derived from the bending measurements, whereas tension measurements results tended to be larger than bending, but smaller than compression. Tensional results were previously shown to be higher than compressional values (Barak et al.^[^ ^88^ ^]^), but we could not verify this.

In 2018 Wu et al.^[10]^ investigated the trabecular bone with different test methods. Their explanation for the wide range of *E* values was on the one hand that some specimens came from healthy and others came from diseased donors, and, on the other hand, methodology related limitations (like the bone specimen hierarchical structure and heterogeneous material properties). In our work, only nondiseased subjects were included, and subgrouping was made by several variables.

For nanoindentation measurements, there are at least eight methods to calculate the *E* value, of which the Oliver‐Pharr method is the most popular. However, the method has limitations as explained by Lewis and Nyman.^[^ ^89^ ^]^ We assume that acquiring higher *E* values by nanoindentation is the consequence of the lack of validation with bone materials. However, the included articles in our study presented no bone validation in the case of nanoindentation measurements.^[^ ^90^ ^]^

Many methodology papers discuss the possible research designs for optimal selection of variable level combinations to achieve better resource management.^[^ ^91^ ^]^ For future studies, we would like to make certain recommendations according to our impact of variables (Fig. 8), cortical and cancellous samples must be considered separate entities. The samples must be wet (saline solution) without freezing or other conservation technique. For sample fixation, samples below the micrometer scale should be fixated by the end‐cap technique.^[^ ^71^ ^]^ The tests have to be done from the same specimens, from three different coordinated axes, with nanoindentation, bending, compression and US testing. For specimen selection, the same age, sex, bone and location matching should be used. Another highly important recommendation is publishing raw individual measurement data, not aggregates or means or figures. With these, the required standardization could be achieved. we were unable to make recommendations as per the optimal testing machine. When preparing FE models, for the selection of the appropriate Young's modulus results, the following parameters must be considered, with decreasing importance macrostructure, measurement method, region of bone, bone, specimen size, orientation, specimen condition, age, and sex.

### Strengths and limitations

Our study has several strengths and limitations. Regarding the strengths, we followed a strict methodology to be transparent and to create a precise and detailed data collection. We managed to include a high number of samples from a high number of publications. This may result in the generalizability of our results.

As for the limitations of this work, the included studies showed a heterogeneous dataset with heterogeneous reporting. First, the reported data were inaccurate, with numerous missing data. Second, we managed to investigate a low number of subgroups. Third, the differences in specimen preparation further increased the heterogeneity. Articles failed to report the time between preparation and testing. Details about the load orientation were sometimes missing. In the case of compression technique, we did not distinguish between platen and end‐cap techniques.

## Implications for Research and Practice

It has been proved that the immediate use of scientific results may have benefits in healthcare.^[^ ^92^, ^93^ ^]^

On the basis of our results more standardized measurement parameters would be advised depending on the parameters and current FE models should take these findings into account. When selecting Young's modulus values one must consider (in decreasing importance): macrostructure, measurement method, region of bone, bone, specimen size, orientation, specimen condition, age, and sex.

In the service of new implant design, our data could help create more accurate FE models, with the consideration of anisotropy and differences of *E* values among the regions within same bone. Most important, our results could help set up more standardized measurement throughout the literature and study designs that allow us to uncover the type and magnitude of the dependence of Young's modulus on the various explanatory variables.

## Conclusion

In summary, we determined the impact weight of the influencing variables on Young's modulus of bone. Significant differences were found in macrostructure, anisotropy, between the femoral epiphysis and metaphysis and in the epiphysis of distal femur and proximal tibia.

## Author Contributions

**Krisztián Kovács:** Conceptualization; formal analysis; methodology; project administration; writing – original draft. **Szilárd Váncsa:** Conceptualization; formal analysis; visualization; writing – review and editing. **Gergely Agócs:** Conceptualization; formal analysis; writing – review and editing. **Andrea Harnos:** Conceptualization; formal analysis; writing – review and editing. **Péter Hegyi:** Conceptualization; funding acquisition; writing – review and editing. **Viktor Weninger:** Conceptualization; writing – review and editing. **Katinka Baross:** Conceptualization; data curation; writing – review and editing. **Bence Kovács:** Conceptualization; data curation; writing – review and editing. **Gergely Soós:** Conceptualization; data curation; writing – review and editing. **György Kocsis:** Conceptualization; supervision; writing – original draft.

## Disclosures

The authors declare no conflicts of interest.

### Peer Review

The peer review history for this article is available at https://www.webofscience.com/api/gateway/wos/peer‐review/10.1002/jbm4.10835.

## Supporting information


**DATA S1.** Supporting Information.
Tables S1–S4.

## Data Availability

The data that support the findings of this study are available from the corresponding author, [GK], upon reasonable request.
