# Supplementary material for: Anisotropy, Anatomical Region, and Additional Variables Influence Young's Modulus of Bone: A Systematic Review and Meta‐Analysis
Source: JBMR Plus. 2023 Oct 31;7(12):e10835. doi: 10.1002/jbm4.10835 (PMC10731124; doi:10.1002/jbm4.10835)
Supplement: Supplementary file 1 — DATA S1. Supporting Information. Tables S1–S4. [file JBM4-7-e10835-s001.docx]

**Supplementary Materials**

**Title:**

Anisotropy, anatomical region and additional variables influence the Young’s modulus of bone: A systematic review and meta-analysis

**Authors**

Krisztián Kovács^1,2^, Szilárd Váncsa^2,3,4^, Gergely Agócs^2,5^, Andrea Harnos^2,6^, Péter Hegyi^2,3,4^, Viktor Weninger^1,2^, Katinka Baross^2^, Bence Kovács^2^, Gergely Soós^2^, György Kocsis^1,2^

**Affiliations:**

1. Department of Orthopaedics, Semmelweis University, Budapest, Hungary
2. Centre for Translational Medicine, Semmelweis University, Budapest, Hungary
3. Institute for Translational Medicine, Szentágothai Research Centre, Medical School, University of Pécs, Pécs, Hungary
4. Division of Pancreatic Diseases, Heart and Vascular Center, Semmelweis University, Budapest, Hungary
5. Department of Biophysics and Radiation Biology, Semmelweis University, Budapest, Hungary
6. Department of Biostatistics, University of Veterinary Medicine, Budapest, Budapest, Hungary

**Corresponding author**

György Kocsis MD, PhD

Postal address: HU-1182, Budapest, Üllői út 78/A, Hungary

Tel.: +36206663522

E-mail address: [kocsisgyorgy@hotmail.com](mailto:kocsisgyorgy@hotmail.com)

**TABLE OF CONTENT**

**Table_S1**. – PRISMA checklist

**Table_S2**. – Summary of results from each included study

**Table_S3**. – Risk of bias assessment using the CARE tool

**Table_S4.** – Supplementary table unfavorable data structure example in case of femur diaphysis

**Table_S1.** PRISMA 2020 checklist. (Page, M.J., et al., *The PRISMA 2020 statement: an updated guideline for reporting systematic reviews.* Bmj, 2021. **372**: p. n71.)

| **Section and topic** | **Item #** | **Checklist item** | **Page where item is reported** |
| --- | --- | --- | --- |
| **Title** | | | |
| Title | 1 | Identify the report as a systematic review. | 1 |
| **Abstract** | | | |
| Abstract | 2 | See the PRISMA 2020 for Abstracts checklist | 3 |
| **Introduction** | | | |
| Rationale | 3 | Describe the rationale for the review in the context of existing knowledge. | 4 |
| Objectives | 4 | Provide an explicit statement of the objective(s) or question(s) the review addresses. | 4 |
| **Methods** | | | |
| Eligibility criteria | 5 | Specify the inclusion and exclusion criteria for the review and how studies were grouped for the syntheses. | 5-6 |
| Information sources | 6 | Specify all databases, registers, websites, organisations, reference lists and other sources searched or consulted to identify studies. Specify the date when each source was last searched or consulted. | 5-6 |
| Search strategy | 7 | Present the full search strategies for all databases, registers and websites, including any filters and limits used. | 5-6 |
| Selection process | 8 | Specify the methods used to decide whether a study met the inclusion criteria of the review, including how many reviewers screened each record and each report retrieved, whether they worked independently, and if applicable, details of automation tools used in the process. | 5-7 |
| Data collection process | 9 | Specify the methods used to collect data from reports, including how many reviewers collected data from each report, whether they worked independently, any processes for obtaining or confirming data from study investigators, and if applicable, details of automation tools used in the process. | 5-7 |
| Data items | 10a | List and define all outcomes for which data were sought. Specify whether all results that were compatible with each outcome domain in each study were sought (e.g. for all measures, time points, analyses), and if not, the methods used to decide which results to collect. | 5-7 |
|  | 10b | List and define all other variables for which data were sought (e.g. participant and intervention characteristics, funding sources). Describe any assumptions made about any missing or unclear information. | 7 |
| Study risk of bias assessment | 11 | Specify the methods used to assess risk of bias in the included studies, including details of the tool(s) used, how many reviewers assessed each study and whether they worked independently, and if applicable, details of automation tools used in the process. | 6; 8 |
| Effect measures | 12 | Specify for each outcome the effect measure(s) (e.g. risk ratio, mean difference) used in the synthesis or presentation of results. | 7-8 |
| Synthesis methods | 13a | Describe the processes used to decide which studies were eligible for each synthesis (e.g. tabulating the study intervention characteristics and comparing against the planned groups for each synthesis (item #5)). | 5-6 |
|  | 13b | Describe any methods required to prepare the data for presentation or synthesis, such as handling of missing summary statistics, or data conversions. | 5-6 |
|  | 13c | Describe any methods used to tabulate or visually display results of individual studies and syntheses. | 7 |
|  | 13d | Describe any methods used to synthesise results and provide a rationale for the choice(s). If meta-analysis was performed, describe the model(s), method(s) to identify the presence and extent of statistical heterogeneity, and software package(s) used. | 5-7 |
|  | 13e | Describe any methods used to explore possible causes of heterogeneity among study results (e.g. subgroup analysis, meta-regression). | 6 |
|  | 13f | Describe any sensitivity analyses conducted to assess robustness of the synthesised results. | 6 |
| Reporting bias assessment | 14 | Describe any methods used to assess risk of bias due to missing results in a synthesis (arising from reporting biases). | 6 |
| Certainty assessment | 15 | Describe any methods used to assess certainty (or confidence) in the body of evidence for an outcome. | 6 |
| **Results** | | | |
| Study selection | 16a | Describe the results of the search and selection process, from the number of records identified in the search to the number of studies included in the review, ideally using a flow diagram. | 7 |
|  | 16b | Cite studies that might appear to meet the inclusion criteria, but which were excluded, and explain why they were excluded. | 7 |
| Study characteristics | 17 | Cite each included study and present its characteristics. | 7 |
| Risk of bias in studies | 18 | Present assessments of risk of bias for each included study. | 8 |
| Results of individual studies | 19 | For all outcomes, present, for each study: (a) summary statistics for each group (where appropriate) and (b) an effect estimate and its precision (e.g. confidence/credible interval), ideally using structured tables or plots. | 7-8 |
| Results of syntheses | 20a | For each synthesis, briefly summarise the characteristics and risk of bias among contributing studies. | 7-8 |
|  | 20b | Present results of all statistical syntheses conducted. If meta-analysis was done, present for each the summary estimate and its precision (e.g. confidence/credible interval) and measures of statistical heterogeneity. If comparing groups, describe the direction of the effect. | 7-8 |
|  | 20c | Present results of all investigations of possible causes of heterogeneity among study results. | 7-8 |
|  | 20d | Present results of all sensitivity analyses conducted to assess the robustness of the synthesised results. | 7-8 |
| Reporting biases | 21 | Present assessments of risk of bias due to missing results (arising from reporting biases) for each synthesis assessed. | 7-8 |
| Certainty of evidence | 22 | Present assessments of certainty (or confidence) in the body of evidence for each outcome assessed. | 7-8 |
| **Discussion** | | | |
| Discussion | 23a | Provide a general interpretation of the results in the context of other evidence. | 9-11 |
|  | 23b | Discuss any limitations of the evidence included in the review. | 9-11 |
|  | 23c | Discuss any limitations of the review processes used. | 9-11 |
|  | 23d | Discuss implications of the results for practice, policy, and future research. | 11 |
| **Additional elements** | | | |
| Registration and protocol | 24a | Provide registration information for the review, including register name and registration number, or state that the review was not registered. | 5 |
|  | 24b | Indicate where the review protocol can be accessed, or state that a protocol was not prepared. | 5 |
|  | 24c | Describe and explain any amendments to information provided at registration or in the protocol. | 5 |
| Support | 25 | Describe sources of financial or non-financial support for the review, and the role of the funders or sponsors in the review. | 11 |
| Competing interests | 26 | Declare any competing interests of review authors. | 11 |
| Availability of data, code, and other materials | 27 | Report which of the following are publicly available and where they can be found: template data collection forms; data extracted from included studies; data used for all analyses; analytic code; any other materials used in the review. | 5-7 |

**Table_S2**. Subgroups comparisons results of the included studies.[1-48]

| **Is there a difference between age groups(<40 vs >60) ?** | | | | | | | | | | | | | | | |
| --- | --- | --- | --- | --- | --- | --- | --- | --- | --- | --- | --- | --- | --- | --- | --- |
| **Femur diaphysis, cortical** | | | | | | |  |  |  |  |  |  |  |  |  |
| **No.** | **Age** | **Anisotropy** | **Sex** | **Condition** | **Size** | **Measuring method** | **Group 1 YM** | **SD** | **Gr 1 count (n)** | **Group 2 YM** | **SD** | **Gr 2 count (n)** | **Mean difference** | ***p*** | ***Article count*** |
|  | <40 vs >60 | axial vs AP vs ML | male vs female | dry vs wet | <1mm vs >1mm | bending vs strain vs compression  vs nanoindentation vs US | **<40** |  |  | **>60** |  |  |  |  |  |
| 1 | NA | - | - | - | - | - | 14.57 | 9.21 | 55 | 19.35 | 3.72 | 74 | 4.77 | *0.001* | 13 |
| 2 | NA | axial | - | - | - | - | 19.18 | 3.24 | 34 | 17.44 | 3.31 | 20 | -1.73 | *0.068* | 6 |
| 3 | NA | AP | - | - | - | - | NA |  |  |  |  |  |  |  |  |
| 4 | NA | ML | - | - | - | - | NA |  |  |  |  |  |  |  |  |
| 5 | NA | - | male | - | - | - | 15.23 | 9.72 | 39 | 21.21 | 4.07 | 29 | 5.97 | *0.001* | 11 |
| 6 | NA | - | female | - | - | - | NA |  |  |  |  |  |  |  |  |
| 7 | NA | - | - | wet | - | - | 13.34 | 9.20 | 48 | 18.05 | 3.30 | 51 | 4.71 | *0.001* | 10 |
| 8 | NA | - | - | dry | - | - | NA |  |  |  |  |  |  |  |  |
| 9 | NA | - | - | - | <1mm | - | NA |  |  |  |  |  |  |  |  |
| 10 | NA | - | - | - | >1mm | - | 14.76 | 9.19 | 54 | 19.35 | 3.72 | 74 | 4.59 | *0.001* | 12 |
| 11 | NA | - | - | - | - | bending | 0.47 | 1.14 | 15 | 18.80 | 3.34 | 31 | 18.33 | *0.000* | 4 |
| 12 | NA | - | - | - | - | tension | NA |  |  |  |  |  |  |  |  |
| 13 | NA | - | - | - | - | compression | NA |  |  |  |  |  |  |  |  |
| 14 | NA | - | - | - | - | nanoindentation | NA |  |  |  |  |  |  |  |  |
| 15 | NA | - | - | - | - | ultrasound | NA |  |  |  |  |  |  |  |  |
| 16 | NA | - | - | wet | >1mm | - | 13.53 | 9.21 | 47 | 18.05 | 3.30 | 51 | 4.52 | *0.002* | 10 |
| 17 | NA | - | male | wet | >1mm | - | NA |  |  |  |  |  |  |  |  |
| 18 | NA | - | male | - | - | bending | NA |  |  |  |  |  |  |  |  |
| 19 | NA | - | male | wet | - | bending | NA |  |  |  |  |  |  |  |  |
| 20 | NA | - | male | wet | >1mm | bending | NA |  |  |  |  |  |  |  |  |
| 21 | NA | axial | male | - | - | - | NA |  |  |  |  |  |  |  |  |
| 22 | NA | axial | - | wet | - | - | 19.19 | 3.29 | 33 | 17.06 | 2.92 | 19 | -2.13 | *0.020* | 5 |
| 23 | NA | axial | male | wet | - | - | NA |  |  |  |  |  |  |  |  |
| 24 | NA | axial | - | - | >1mm | - | 19.18 | 3.24 | 34 | 17.443 | 3.31 | 20 | -1.73 | *0.068* | 6 |
| 25 | NA | axial | - | wet | >1mm | - | 19.19 | 3.29 | 33 | 17.06 | 2.92 | 19 | -2.13 | *0.020* | 5 |
| 26 | NA | axial | male | wet | >1mm | - | NA |  |  |  |  |  |  |  |  |
| 27 | NA | axial | - | - | - | bending | NA |  |  |  |  |  |  |  |  |
| 28 | NA | axial | male | - | - | bending | NA |  |  |  |  |  |  |  |  |
| 29 | NA | axial | male | wet | - | bending | NA |  |  |  |  |  |  |  |  |
| 30 | NA | axial | male | wet | >1mm | bending | NA |  |  |  |  |  |  |  |  |
| 31 | NA | AP | male | - | - | - | NA |  |  |  |  |  |  |  |  |
| 32 | NA | AP | - | wet | - | - | NA |  |  |  |  |  |  |  |  |
| 33 | NA | AP | male | wet | - | - | NA |  |  |  |  |  |  |  |  |
| 34 | NA | AP | - | - | >1mm | - | NA |  |  |  |  |  |  |  |  |
| 35 | NA | AP | - | wet | >1mm | - | NA |  |  |  |  |  |  |  |  |
| 36 | NA | AP | male | wet | >1mm | - | NA |  |  |  |  |  |  |  |  |
| 37 | NA | AP | - | - | - | bending | NA |  |  |  |  |  |  |  |  |
| 38 | NA | AP | - | wet | - | bending | NA |  |  |  |  |  |  |  |  |
| 39 | NA | AP | male | - | - | bending | NA |  |  |  |  |  |  |  |  |
| 40 | NA | AP | male | wet | - | bending | NA |  |  |  |  |  |  |  |  |
| 41 | NA | AP | male | wet | >1mm | bending | NA |  |  |  |  |  |  |  |  |
|  | | | | | | |  | | | | | | | | |
| **Fibula diaphysis, cortical** | | | | | | |  | | | | | | | | |
| **No.** | **Age** | **Anisotropy** | **Sex** | **Condition** | **Size** | **Measuring method** | **Group 1 YM** | **SD** | **Gr 1 count (n)** | **Group 2 YM** | **SD** | **Gr 2 count (n)** | **Mean difference** | ***p*** | **Article count** |
|  | <40 vs >60 | axial vs AP vs ML | male vs female | dry vs wet | <1mm vs >1mm | bending vs strain vs compression  vs nanoindentation vs US | **<40** |  |  | **>60** |  |  |  |  |  |
| 42 | NA | - | - | - | - | - | 14.18 | 4.16 | 11 | 14.21 | 4.51 | 24 | 0.02 | *0.988* | 2 |
| 43 | NA | axial | - | - | - | - | NA |  |  |  |  |  |  |  |  |
| 44 | NA | AP | - | - | - | - | NA |  |  |  |  |  |  |  |  |
| 45 | NA | ML | - | - | - | - | NA |  |  |  |  |  |  |  |  |
| 46 | NA | - | male | - | - | - | NA |  |  |  |  |  |  |  |  |
| 47 | NA | - | female | - | - | - | NA |  |  |  |  |  |  |  |  |
| 48 | NA | - | - | wet | - | - | 14.18 | 4.16 | 11 | 14.21 | 4.51 | 24 | 0.02 | *0.988* | 2 |
| 49 | NA | - | - | dry | - | - | NA |  |  |  |  |  |  |  |  |
| 50 | NA | - | - | - | <1mm | - | NA |  |  |  |  |  |  |  |  |
| 51 | NA | - | - | - | >1mm | - | 14.18 | 4.16 | 11 | 13.32 | 4.45 | 13 | -0.87 | *0.626* | 2 |
| 52 | NA | - | - | - | - | bending | NA |  |  |  |  |  |  |  |  |
| 53 | NA | - | - | - | - | tension | NA |  |  |  |  |  |  |  |  |
| 54 | NA | - | - | - | - | compression | NA |  |  |  |  |  |  |  |  |
| 55 | NA | - | - | - | - | nanoidentitation | NA |  |  |  |  |  |  |  |  |
| 56 | NA | - | - | - | - | ultrasound | NA |  |  |  |  |  |  |  |  |
| 57 | NA | - | - | wet | >1mm | - | 14.18 | 4.16 | 11 | 13.32 | 4.45 | 13 | -0.87 | *0.626* | 2 |
|  |  |  |  |  |  |  |  |  |  |  |  |  |  |  |  |
| **Is there a difference between loading axises (axial vs AP vs ML) ?** | | | | | | | | | | | | | | | |
| **Femur diaphysis, cortical** | | | | | | |  | | | | | | | | |
| **No.** | **Age** | **Anisotropy** | **Sex** | **Condition** | **Size** | **Measuring method** | **Group 1 YM** | **SD** | **Gr 1 count (n)** | **Group 2 YM** | **SD** | **Gr 2 count (n)** | **Mean difference** | ***p*** | **Article count** |
|  | <40 vs >60 | axial vs AP vs ML | male vs female | dry vs wet | <1mm vs >1mm | bending vs strain vs compression  vs nanoindentation vs US | **AXIAL** |  |  | **AP** |  |  |  |  |  |
| 58 | - | NA | - | - | - | - | 18.87 | 3.09 | 135 | 10.46 | 1.98 | 27 | -8.42 | *0.000* | 11 |
| 59 | <40 | NA | - | - | - | - | NA |  |  |  |  |  |  |  |  |
| 60 | >60 | NA | - | - | - | - | NA |  |  |  |  |  |  |  |  |
| 61 | - | NA | male | - | - | - | NA |  |  |  |  |  |  |  |  |
| 62 | - | NA | female | - | - | - | NA |  |  |  |  |  |  |  |  |
| 63 | - | NA | - | - | >1mm | - | 17.86 | 3.05 | 89 | 10.10 | 1.98 | 27 | -7.54 | *0.000* | 10 |
| 64 | - | NA | - | - | <1mm | - | NA |  |  |  |  |  |  |  |  |
| 65 | - | NA | - | wet | - | - | NA |  |  |  |  |  |  |  |  |
| 66 | - | NA | - | dry | - | - | NA |  |  |  |  |  |  |  |  |
| 67 | - | NA | - | - | - | nanoindentation | NA |  |  |  |  |  |  |  |  |
| 68 | - | NA | - | - | - | compression | NA |  |  |  |  |  |  |  |  |
| 69 | - | NA | - | - | - | ultrasound | NA |  |  |  |  |  |  |  |  |
| 70 | - | NA | - | - | - | bending | NA |  |  |  |  |  |  |  |  |
| 71 | - | NA | - | - | - | tension | 17.46 | 2.89 | 57 | 9.55 | 3.30 | 18 | -7.91 | *0.000* | 7 |
| 72 | - | NA | - | wet | <1mm | - | NA |  |  |  |  |  |  |  |  |
| 73 | - | NA | - | dry | <1mm | - | NA |  |  |  |  |  |  |  |  |
| 74 | - | NA | - | wet | >1mm | - | NA |  |  |  |  |  |  |  |  |
| 75 | - | NA | - | dry | >1mm | - | NA |  |  |  |  |  |  |  |  |
| 76 | - | NA | - | wet | <1mm | tension | NA |  |  |  |  |  |  |  |  |
| 77 | - | NA | - | dry | <1mm | tension | NA |  |  |  |  |  |  |  |  |
| 78 | - | NA | - | wet | >1mm | tension | NA |  |  |  |  |  |  |  |  |
| 79 | - | NA | - | dry | >1mm | tension | NA |  |  |  |  |  |  |  |  |
|  | | | | | | |  | | | | | | | | |
| **No.** | **Age** | **Anisotropy** | **Sex** | **Condition** | **Size** | **Measuring method** | **Group 1 YM** | **SD** | **Gr 1 count (n)** | **Group 2 YM** | **SD** | **Gr 2 count (n)** | **Mean difference** | ***p*** | **Article count** |
|  | <40 vs >60 | axial vs AP vs ML | male vs female | dry vs wet | <1mm vs >1mm | bending vs strain vs compression  vs nanoindentation vs US | **AP** |  |  | **ML** |  |  |  |  |  |
| 80 | - | NA | - | - | - | - | NA |  |  |  |  |  |  |  |  |
|  |  |  |  |  |  |  | **AXIAL** |  |  | **ML** |  |  |  |  |  |
| 81 | - | NA | - | - | - | - | NA |  |  |  |  |  |  |  |  |
|  |  |  |  |  |  |  |  |  |  |  |  |  |  |  |  |
| **Is there a difference between sample conditions (dry vs wet) ?** | | | | | | | | | | | | | | | |
| **Femur diaphysis, cortical** | | | | | | |  | | | | | | | | |
| **No.** | **Age** | **Anisotropy** | **Sex** | **Condition** | **Size** | **Measuring method** | **Group 1 YM** | **SD** | **Gr 1 count (n)** | **Group 2 YM** | **SD** | **Gr 2 count (n)** | **Mean difference** | ***p*** | **Article count** |
|  | <40 vs >60 | axial vs AP vs ML | male vs female | dry vs wet | <1mm vs >1mm | bending vs strain vs compression  vs nanoindentation vs US | **Dry** |  |  | **Wet** |  |  |  |  |  |
| 82 | - | - | - | NA | - | - | 22.42 | 2.36 | 61 | 16.88 | 6.33 | 178 | -5.54 | *0.000* | 18 |
| 83 | - | axial | - | NA | - | - | NA |  |  |  |  |  |  |  |  |
| 84 | - | AP | - | NA | - | - | NA |  |  |  |  |  |  |  |  |
| 85 | - | ML | - | NA | - | - | NA |  |  |  |  |  |  |  |  |
| 86 | - | - | female | NA | - | - | NA |  |  |  |  |  |  |  |  |
| 87 | - | - | male | NA | - | - | 22.48 | 2.33 | 60 | 17.30 | 6.87 | 104 | -5.18 | *0.000* | 14 |
| 88 | <40 | - | - | NA | - | - | NA |  |  |  |  |  |  |  |  |
| 89 | >60 | - | - | NA | - | - | 22.22 | 2.92 | 23 | 18.05 | 3.30 | 51 | -4.18 | *0.000* | 8 |
| 90 | - | - | - | NA | <1mm | - | NA |  |  |  |  |  |  |  |  |
| 91 | - | - | - | NA | >1mm | - | 22.42 | 2.36 | 61 | 15.91 | 6.49 | 129 | -6.51 | *0.000* | 15 |
| 92 | - | - | - | NA | - | nanoindentation | 22.59 | 2.20 | 59 | 20.58 | 2.40 | 46 | -2.01 | *0.000* | 4 |
| 93 | - | - | - | NA | - | compression | NA |  |  |  |  |  |  |  |  |
| 94 | - | - | - | NA | - | ultrasound | NA |  |  |  |  |  |  |  |  |
| 95 | - | - | - | NA | - | bending | NA |  |  |  |  |  |  |  |  |
| 96 | - | - | - | NA | - | tension | NA |  |  |  |  |  |  |  |  |
| 97 | <40 | - | male | NA | - | - | NA |  |  |  |  |  |  |  |  |
| 98 | >60 | - | male | NA | - | - | NA |  |  |  |  |  |  |  |  |
| 99 | - | - | male | NA | >1mm | - | 22.48 | 2.33 | 60 | 14.87 | 8.03 | 57 | -7.61 | *0.000* | 12 |
| 100 | - | - | male | NA | - | nanoindentation | 22.59 | 2.20 | 59 | 20.58 | 2.40 | 46 | -2.01 | *0.000* | 4 |
| 101 | >60 | - | - | NA | >1mm | - | 22.22 | 2.92 | 23 | 18.05 | 3.30 | 51 | -4.18 | *0.000* | 8 |
| 102 | >60 | - | - | NA | - | nanoindentation | NA |  |  |  |  |  |  |  |  |
| 103 | - | - | - | NA | >1mm | nanoindentation | NA |  |  |  |  |  |  |  |  |
|  |  |  |  |  |  |  |  |  |  |  |  |  |  |  |  |
| **Is there a difference between male and female ?** | | | | | | | | | | | | | | | |
| **Femur diaphysis, cortical** | | | | | | |  | | | | | | | | |
| **No.** | **Age** | **Anisotropy** | **Sex** | **Condition** | **Size** | **Measuring method** | **Group 1 YM** | **SD** | **Gr 1 count (n)** | **Group 2 YM** | **SD** | **Gr 2 count (n)** | **Mean difference** | ***p*** | **Article count** |
|  | <40 vs >60 | axial vs AP vs ML | male vs female | dry vs wet | <1mm vs >1mm | bending vs strain vs compression  vs nanoindentation vs US | **Male** |  |  | **Female** |  |  |  |  |  |
| 104 | - | - | NA | - | - | - | 19.20 | 6.17 | 164 | 11.25 | 7.36 | 15 | 7.95 | *0.001* | 16 |
| 105 | - | axial | NA | - | - | - | NA |  |  |  |  |  |  |  |  |
| 106 | - | AP | NA | - | - | - | NA |  |  |  |  |  |  |  |  |
| 107 | - | ML | NA | - | - | - | NA |  |  |  |  |  |  |  |  |
| 108 | <40 | - | NA | - | - | - | NA |  |  |  |  |  |  |  |  |
| 109 | >60 | - | NA | - | - | - | NA |  |  |  |  |  |  |  |  |
| 110 | - | - | NA | - | <1mm | - | NA |  |  |  |  |  |  |  |  |
| 111 | - | - | NA | - | >1mm | - | 18.78 | 6.97 | 117 | 11.25 | 7.36 | 15 | 7.53 | *0.002* | 14 |
| 112 | - | - | NA | wet | - | - | 17.30 | 6.87 | 104 | 10.71 | 7.33 | 14 | 6.59 | *0.006* | 12 |
| 113 | - | - | NA | dry | - | - | NA |  |  |  |  |  |  |  |  |
| 114 | - | - | NA | - | - | bending | 5.84 | 7.20 | 18 | 9.57 | 7.93 | 11 | -3.73 | *0.219* | 4 |
| 115 | - | - | NA | - | - | tension | NA |  |  |  |  |  |  |  |  |
| 116 | - | - | NA | - | - | compression | NA |  |  |  |  |  |  |  |  |
| 117 | - | - | NA | - | - | nanoindentation | NA |  |  |  |  |  |  |  |  |
| 118 | - | - | NA | - | - | ultrasound | NA |  |  |  |  |  |  |  |  |
| 119 | - | - | NA | wet | >1mm | - | 14.87 | 8.03 | 57 | 10.71 | 7.33 | 14 | 4.16 | *0.076* | 10 |
| 120 | - | - | NA | wet | - | bending | 5.84 | 7.20 | 18 | 9.57 | 7.93 | 11 | -3.73 | *0.219* | 4 |
| 121 | - | - | NA | - | >1mm | bending | 5.92 | 7.41 | 17 | 9.57 | 7.93 | 11 | -3.65 | *0.236* | 3 |
| 122 | - | - | NA | wet | >1mm | bending | 5.92 | 7.41 | 17 | 9.57 | 7.93 | 11 | -3.65 | *0.236* | 3 |
|  | | | | | | |  | | | | | | | | |
| **Femur epiphysis, cancellous** | | | | | | |  | | | | | | | | |
| **No.** | **Age** | **Anisotropy** | **Sex** | **Condition** | **Size** | **Measuring method** | **Group 1 YM** | **SD** | **Gr 1 count (n)** | **Group 2 YM** | **SD** | **Gr 2 count (n)** | **Mean difference** | ***p*** | **Article count** |
|  | <40 vs >60 | axial vs AP vs ML | male vs female | dry vs wet | <1mm vs >1mm | bending vs strain vs compression  vs nanoindentation vs US | **Male** |  |  | **Female** |  |  |  |  |  |
| 123 | - | - | NA | - | - | - | 0.63 | 0.50 | 65 | 3.38 | 6.56 | 11 | 2.75 | *0.195* | 5 |
| 124 | - | axial | NA | - | - | - | NA |  |  |  |  |  |  |  |  |
| 125 | - | AP | NA | - | - | - | NA |  |  |  |  |  |  |  |  |
| 126 | - | ML | NA | - | - | - | NA |  |  |  |  |  |  |  |  |
| 127 | <40 | - | NA | - | - | - | NA |  |  |  |  |  |  |  |  |
| 128 | >60 | - | NA | - | - | - | NA |  |  |  |  |  |  |  |  |
| 129 | - | - | NA | - | <1mm | - | NA |  |  |  |  |  |  |  |  |
| 130 | - | - | NA | - | >1mm | - | 0.63 | 0.50 | 65 | 3.38 | 6.56 | 11 | 2.75 | *0.195* | 5 |
| 131 | - | - | NA | wet | - | - | 0.63 | 0.50 | 65 | 1.47 | 1.79 | 10 | 0.84 | *0.174* | 4 |
| 132 | - | - | NA | dry | - | - | NA |  |  |  |  |  |  |  |  |
| 133 | - | - | NA | - | - | bending | NA |  |  |  |  |  |  |  |  |
| 134 | - | - | NA | - | - | tension | NA |  |  |  |  |  |  |  |  |
| 135 | - | - | NA | - | - | compression | NA |  |  |  |  |  |  |  |  |
| 136 | - | - | NA | - | - | nanoidentitation | NA |  |  |  |  |  |  |  |  |
| 137 | - | - | NA | - | - | ultrasound | NA |  |  |  |  |  |  |  |  |
| 138 | - | axial | NA | - | >1mm | - | NA |  |  |  |  |  |  |  |  |
| 139 | - | - | NA | wet | >1mm | - | 0.63 | 0.50 | 65 | 1.47 | 1.79 | 10 | 0.84 | *0.174* | 4 |
| 140 | - | - | NA | - | >1mm | compression | NA |  |  |  |  |  |  |  |  |
| 141 | - | axial | NA | wet | >1mm | - | NA |  |  |  |  |  |  |  |  |
| 142 | - | axial | NA | - | >1mm | compression | NA |  |  |  |  |  |  |  |  |
| 143 | - | - | NA | wet | >1mm | compression | NA |  |  |  |  |  |  |  |  |
| 144 | - | axial | NA | wet | >1mm | compression | NA |  |  |  |  |  |  |  |  |
|  | | | | | | |  | | | | | | | | |
| **Fibula diaphysis, cortical** | | | | | | |  | | | | | | | | |
| **No.** | **Age** | **Anisotropy** | **Sex** | **Condition** | **Size** | **Measuring method** | **Group 1 YM** | **SD** | **Gr 1 count (n)** | **Group 2 YM** | **SD** | **Gr 2 count (n)** | **Mean difference** | ***p*** | **Article count** |
|  | <40 vs >60 | axial vs AP vs ML | male vs female | dry vs wet | <1mm vs >1mm | bending vs strain vs compression  vs nanoindentation vs US | **Male** |  |  | **Female** |  |  |  |  |  |
| 145 | - | - | NA | - | - | - | 13.94 | 4.29 | 25 | 14.86 | 4.39 | 11 | 0.93 | *0.564* | 2 |
| 146 | - | axial | NA | - | - | - | NA |  |  |  |  |  |  |  |  |
| 147 | - | AP | NA | - | - | - | NA |  |  |  |  |  |  |  |  |
| 148 | - | ML | NA | - | - | - | NA |  |  |  |  |  |  |  |  |
| 149 | <40 | - | NA | - | - | - | NA |  |  |  |  |  |  |  |  |
| 150 | >60 | - | NA | - | - | - | NA |  |  |  |  |  |  |  |  |
| 151 | - | - | NA | - | <1mm | - | NA |  |  |  |  |  |  |  |  |
| 152 | - | - | NA | - | >1mm | - | NA |  |  |  |  |  |  |  |  |
| 153 | - | - | NA | wet | - | - | 13.94 | 4.29 | 25 | 14.86 | 4.39 | 11 | 0.93 | *0.564* | 2 |
| 154 | - | - | NA | dry | - | - | NA |  |  |  |  |  |  |  |  |
| 155 | - | - | NA | - | - | bending | NA |  |  |  |  |  |  |  |  |
| 156 | - | - | NA | - | - | tension | NA |  |  |  |  |  |  |  |  |
| 157 | - | - | NA | - | - | compression | NA |  |  |  |  |  |  |  |  |
| 158 | - | - | NA | - | - | nanoidentitation | NA |  |  |  |  |  |  |  |  |
| 159 | - | - | NA | - | - | ultrasound | NA |  |  |  |  |  |  |  |  |
|  |  |  |  |  |  |  |  |  |  |  |  |  |  |  |  |
| **Is there a difference between sample Size (<1mm vs >1mm) ?** | | | | | | | | | | | | | | | |
| **Femur diaphysis, cortical** | | | | | | |  | | | | | | | | |
| **No.** | **Age** | **Anisotropy** | **Sex** | **Condition** | **Size** | **Measuring method** | **Group 1 YM** | **SD** | **Gr 1 count (n)** | **Group 2 YM** | **SD** | **Gr 2 count (n)** | **Mean difference** | ***p*** | **Article count** |
|  | <40 vs >60 | axial vs AP vs ML | male vs female | dry vs wet | <1mm vs >1mm | bending vs strain vs compression  vs nanoindentation vs US | **<1mm** |  |  | **>1mm** |  |  |  |  |  |
| 160 | - | - | - | - | NA | - | 20.24 | 3.33 | 47 | 17.22 | 6.25 | 225 | -3.02 | *0.000* | 18 |
| 161 | - | axial | - | - | NA | - | 20.58 | 2.40 | 46 | 18.00 | 3.05 | 89 | -2.58 | *0.000* | 10 |
| 162 | - | AP | - | - | NA | - | NA |  |  |  |  |  |  |  |  |
| 163 | - | ML | - | - | NA | - | NA |  |  |  |  |  |  |  |  |
| 164 | - | - | female | - | NA | - | NA |  |  |  |  |  |  |  |  |
| 165 | - | - | male | - | NA | - | 20.24 | 3.33 | 47 | 18.78 | 6.97 | 117 | -1.46 | *0.071* | 14 |
| 166 | <40 | - | - | - | NA | - | NA |  |  |  |  |  |  |  |  |
| 167 | >60 | - | - | - | NA | - | NA |  |  |  |  |  |  |  |  |
| 168 | - | - | - | wet | NA | - | 20.24 | 3.33 | 47 | 15.91 | 6.49 | 129 | -4.33 | *0.000* | 13 |
| 169 | - | - | - | dry | NA | - | NA |  |  |  |  |  |  |  |  |
| 170 | - | - | - | - | NA | nanoindentation | 20.58 | 2.40 | 46 | 22.59 | 2.20 | 59 | 02.01 | *0.000* | 4 |
| 171 | - | - | - | - | NA | compression | NA |  |  |  |  |  |  |  |  |
| 172 | - | - | - | - | NA | ultrasound | NA |  |  |  |  |  |  |  |  |
| 173 | - | - | - | - | NA | bending | NA |  |  |  |  |  |  |  |  |
| 174 | - | - | - | - | NA | tension | NA |  |  |  |  |  |  |  |  |
| 175 | - | - | male | wet | NA | - | 20.24 | 3.33 | 47 | 14.87 | 8.03 | 57 | -5.37 | *0.000* | 10 |
| 176 | - | - | male | wet | NA | nanoindentation | NA |  |  |  |  |  |  |  |  |
| 177 | - | axial | male | wet | NA | - | 20.58 | 2.40 | 46 | 20.55 | 3.26 | 28 | -0.03 | *0.969* | 4 |
| 178 | - | axial | male | wet | NA | nanoindentation | NA |  |  |  |  |  |  |  |  |
|  | | | | | | |  | | | | | | | | |
| **Fibula diaphysis, cortical** | | | | | | |  | | | | | | | | |
| **No.** | **Age** | **Anisotropy** | **Sex** | **Condition** | **Size** | **Measuring method** | **Group 1 YM** | **SD** | **Gr 1 count (n)** | **Group 2 YM** | **SD** | **Gr 2 count (n)** | **Mean difference** | ***p*** | **Article count** |
|  | <40 vs >60 | axial vs AP vs ML | male vs female | dry vs wet | <1mm vs >1mm | bending vs strain vs compression  vs nanoindentation vs US | **<1mm** |  |  | **>1mm** |  |  |  |  |  |
| 179 | - | - | - | - | NA | - | 15.26 | 4.56 | 11 | 13.76 | 4.17 | 25 | -1.50 | *0.362* | 2 |
| 180 | - | - | male | - | NA | - | NA |  |  |  |  |  |  |  |  |
| 181 | - | - | female | - | NA | - | NA |  |  |  |  |  |  |  |  |
| 182 | <40 | - | - | - | NA | - | NA |  |  |  |  |  |  |  |  |
| 183 | >60 | - | - | - | NA | - | 15.26 | 4.56 | 11 | 13.32 | 4.45 | 13 | -1.95 | *0.304* | 1 |
| 184 | - | - | - | dry | NA | - | NA |  |  |  |  |  |  |  |  |
| 185 | - | - | - | wet | NA | - | 15.26 | 4.56 | 11 | 13.76 | 4.17 | 25 | -1.50 | *0.362* | 2 |
| 186 | - | - | - | - | NA | tension | NA |  |  |  |  |  |  |  |  |
| 187 | - | - | - | - | NA | compression | NA |  |  |  |  |  |  |  |  |
| 188 | - | - | - | - | NA | bending | NA |  |  |  |  |  |  |  |  |
| 189 | - | - | - | - | NA | nanoidentitation | NA |  |  |  |  |  |  |  |  |
| 190 | - | - | - | - | NA | ultrasound | NA |  |  |  |  |  |  |  |  |
| 191 | - | AP | - | - | NA | - | NA |  |  |  |  |  |  |  |  |
| 192 | - | axial | - | - | NA | - | NA |  |  |  |  |  |  |  |  |
| 193 | - | ML | - | - | NA | - | NA |  |  |  |  |  |  |  |  |
| 194 | >60 | - | - | wet | NA | - | 15.26 | 4.56 | 11 | 13.32 | 4.45 | 13 | -1.95 | *0.304* | 1 |
|  |  |  |  |  |  |  |  |  |  |  |  |  |  |  |  |
| **Is there a difference between methods ?** | | | | | | | | | | | | | | | |
| **Fibula diaphysis, cortical** | | | | | | |  | | | | | | | | |
| **No.** | **Age** | **Anisotropy** | **Sex** | **Condition** | **Size** | **Measuring method** | **Group 1 YM** | **SD** | **Gr 1 count (n)** | **Group 2 YM** | **SD** | **Gr 2 count (n)** | **Mean difference** | ***p*** | **Article count** |
|  | <40 vs >60 | axial vs AP vs ML | male vs female | dry vs wet | <1mm vs >1mm | bending vs strain vs compression  vs nanoindentation vs US | **Tension** |  |  | **Compression** |  |  |  |  |  |
| 195 | - | - | - | - | - | NA | NA |  |  |  |  |  |  |  |  |
|  |  |  |  |  |  |  | **Tension** |  |  | **Bending** |  |  |  |  |  |
| 196 | - | - | - | - | - | NA | NA |  |  |  |  |  |  |  |  |
|  |  |  |  |  |  |  | **Tension** |  |  | **Nanoind.** |  |  |  |  |  |
| 197 | - | - | - | - | - | NA | NA |  |  |  |  |  |  |  |  |
|  |  |  |  |  |  |  | **Tension** |  |  | **Ultrasound** |  |  |  |  |  |
| 198 | - | - | - | - | - | NA | NA |  |  |  |  |  |  |  |  |
|  |  |  |  |  |  |  | **Compression** |  |  | **Nanoind.** |  |  |  |  |  |
| 199 | - | - | - | - | - | NA | NA |  |  |  |  |  |  |  |  |
|  |  |  |  |  |  |  | **Compression** |  |  | **Ultrasound** |  |  |  |  |  |
| 200 | - | - | - | - | - | NA | NA |  |  |  |  |  |  |  |  |
|  |  |  |  |  |  |  | **Nanoind.** |  |  | **Ultrasound** |  |  |  |  |  |
| 201 | - | - | - | - | - | NA | NA |  |  |  |  |  |  |  |  |
|  |  |  |  |  |  |  | **Bending** |  |  | **Compression** |  |  |  |  |  |
| 202 | - | - | - | - | - | NA | NA |  |  |  |  |  |  |  |  |
|  |  |  |  |  |  |  | **Bending** |  |  | **Nanoind.** |  |  |  |  |  |
| 203 | - | - | - | - | - | NA | NA |  |  |  |  |  |  |  |  |
|  |  |  |  |  |  |  | **Bending** |  |  | **Ultrasound** |  |  |  |  |  |
| 204 | - | - | - | - | - | NA | 9.10 | 1.55 | 13 | 17.14 | 1.89 | 21 | 08.04 | *0.000* | 1 |
| 205 | - | axial | - | - | - | NA | NA |  |  |  |  |  |  |  |  |
| 206 | - | AP | - | - | - | NA | NA |  |  |  |  |  |  |  |  |
| 207 | - | ML | - | - | - | NA | NA |  |  |  |  |  |  |  |  |
| 208 | <40 | - | - | - | - | NA | NA |  |  |  |  |  |  |  |  |
| 209 | >60 | - | - | - | - | NA | NA |  |  |  |  |  |  |  |  |
| 210 | - | - | male | - | - | NA | 9.19 | 1.57 | 10 | 17.14 | 1.75 | 13 | 7.95 | *0.000* | 1 |
| 211 | - | - | female | - | - | NA | NA |  |  |  |  |  |  |  |  |
| 212 | - | - | - | wet | - | NA | 9.10 | 1.55 | 13 | 17.14 | 1.89 | 21 | 08.04 | *0.000* | 1 |
| 213 | - | - | - | dry | - | NA | NA |  |  |  |  |  |  |  |  |
| 214 | - | - | - | - | <1mm | NA | NA |  |  |  |  |  |  |  |  |
| 215 | - | - | - | - | >1mm | NA | 9.14 | 1.44 | 10 | 16.84 | 1.69 | 13 | 7.70 | *0.000* | 1 |
| 216 | - | - | male | wet | - | NA | 9.19 | 1.57 | 10 | 17.14 | 1.75 | 13 | 7.95 | *0.000* | 1 |
| 217 | - | - | male | - | >1mm | NA | NA |  |  |  |  |  |  |  |  |
| 218 | - | - | - | wet | >1mm | NA | 9.14 | 1.44 | 10 | 16.84 | 1.69 | 13 | 7.70 | *0.000* | 1 |
| 219 | - | - | male | wet | >1mm | NA |  |  |  |  |  |  |  |  |  |
|  | | | | | | |  | | | | | | | | |
| **Femur diaphysis, cortical** | | | | | | |  | | | | | | | | |
| **No.** | **Age** | **Anisotropy** | **Sex** | **Condition** | **Size** | **Measuring method** | **Group 1 YM** | **SD** | **Gr 1 count (n)** | **Group 2 YM** | **SD** | **Gr 2 count (n)** | **Mean difference** | ***p*** | **Article count** |
|  | <40 vs >60 | axial vs AP vs ML | male vs female | dry vs wet | <1mm vs >1mm | bending vs strain vs compression  vs nanoindentation vs US | **Tension** |  |  | **Ultrasound** |  |  |  |  |  |
| 220 |  |  |  |  |  | NA | NA |  |  |  |  |  |  |  |  |
|  |  |  |  |  |  |  | **Compression** |  |  | **Ultrasound** |  |  |  |  |  |
| 221 |  |  |  |  |  | NA | NA |  |  |  |  |  |  |  |  |
|  |  |  |  |  |  |  | **Bending** |  |  | **Ultrasound** |  |  |  |  |  |
| 222 |  |  |  |  |  | NA | NA |  |  |  |  |  |  |  |  |
|  |  |  |  |  |  |  | **Nanoind.** |  |  | **Ultrasound** |  |  |  |  |  |
| 223 |  |  |  |  |  | NA | NA |  |  |  |  |  |  |  |  |
|  |  |  |  |  |  |  | **Tension** |  |  | **Compression** |  |  |  |  |  |
| 224 | - | - | - | - | - | NA | 15.39 | 4.24 | 87 | 17.93 | 4.14 | 31 | 2.55 | *0.005* | 10 |
| 225 | - | axial | - | - | - | NA | 17.46 | 2.89 | 57 | 18.51 | 2.63 | 30 | 01.05 | *0.091* | 7 |
| 226 | - | AP | - | - | - | NA | NA |  |  |  |  |  |  |  |  |
| 227 | - | ML | - | - | - | NA | NA |  |  |  |  |  |  |  |  |
| 228 | <40 | - | - | - | - | NA | 18.74 | 3.46 | 23 | 20.09 | 2.62 | 11 | 1.35 | *0.218* | 4 |
| 229 | >60 | - | - | - | - | NA | NA |  |  |  |  |  |  |  |  |
| 230 | - | - | male | - | - | NA | 17.44 | 4.49 | 30 | 21.31 | 1.81 | 10 | 3.87 | *0.000* | 3 |
| 231 | - | - | female | - | - | NA | NA |  |  |  |  |  |  |  |  |
| 232 | - | - | - | wet | - | NA | 16.99 | 3.73 | 50 | 17.93 | 1.81 | 31 | 0.94 | *0.305* | 8 |
| 233 | - | - | - | dry | - | NA | NA |  |  |  |  |  |  |  |  |
| 234 | - | - | - | - | <1mm | NA | NA |  |  |  |  |  |  |  |  |
| 235 | - | - | - | - | >1mm | NA | 15.39 | 4.24 | 87 | 18.51 | 1.81 | 30 | 3.13 | *0.000* | 9 |
| 236 | <40 | axial | - | - | - | NA | 18.74 | 3.46 | 23 | 20.09 | 2.62 | 11 | 1.35 | *0.218* | 4 |
| 237 | >60 | axial | - | - | - | NA | NA |  |  |  |  |  |  |  |  |
| 238 | <40 | - | male | - | - | NA | NA |  |  |  |  |  |  |  |  |
| 239 | >60 | - | male | - | - | NA | NA |  |  |  |  |  |  |  |  |
| 240 | <40 | - | female | - | - | NA | NA |  |  |  |  |  |  |  |  |
| 241 | >60 | - | female | - | - | NA | NA |  |  |  |  |  |  |  |  |
| 242 | <40 | - | - | wet | - | NA | 18.74 | 3.54 | 22 | 20.09 | 2.62 | 11 | 1.35 | *0.228* | 4 |
| 243 | >60 | - | - | wet | - | NA | NA |  |  |  |  |  |  |  |  |
| 244 | <40 | - | - | - | >1mm | NA | 18.74 | 3.46 | 23 | 20.09 | 2.62 | 11 | 1.35 | *0.218* | 4 |
| 245 | >60 | - | - | - | >1mm | NA | NA |  |  |  |  |  |  |  |  |
| 246 | <40 | axial | male | - | - | NA | NA |  |  |  |  |  |  |  |  |
| 247 | <40 | axial | - | wet | - | NA | 18.74 | 3.54 | 22 | 20.09 | 2.62 | 11 | 1.35 | *0.228* | 4 |
| 248 | >60 | axial | - | wet | - | NA | NA |  |  |  |  |  |  |  |  |
| 249 | <40 | axial | - | - | >1mm | NA | 18.74 | 3.46 | 23 | 20.09 | 2.62 | 11 | 1.35 | *0.218* | 4 |
| 250 | >60 | axial | - | - | >1mm | NA | NA |  |  |  |  |  |  |  |  |
| 251 | - | axial | male | wet | - | NA | 19.73 | 3.52 | 17 | 21.31 | 1.81 | 10 | 1.58 | *0.138* | 2 |
| 252 | - | axial | female | wet | - | NA | NA |  |  |  |  |  |  |  |  |
| 253 | - | axial | - | wet | >1mm | NA | 17.84 | 3.25 | 38 | 18.51 | 2.63 | 30 | 0.67 | *0.347* | 5 |
| 254 | - | - | male | wet | >1mm | NA | 17.49 | 4.56 | 29 | 21.31 | 1.81 | 10 | 3.82 | *0.001* | 4 |
| 255 | - | - | female | wet | >1mm | NA | NA |  |  |  |  |  |  |  |  |
| 256 | <40 | axial | male | wet | - | NA | 19.53 | 3.53 | 16 | 22.15 | 0.57 | 6 | 2.62 | *0.011* | 2 |
| 257 | <40 | axial | - | wet | >1mm | NA | 18.74 | 3.54 | 22 | 20.09 | 2.62 | 11 | 1.35 | *0.228* | 4 |
| 258 | >60 | axial | - | wet | >1mm | NA | NA |  |  |  |  |  |  |  |  |
| 259 | <40 | axial | male | - | >1mm | NA | NA |  |  |  |  |  |  |  |  |
| 260 | <40 | - | male | wet | >1mm | NA | NA |  |  |  |  |  |  |  |  |
| 261 | - | axial | male | wet | >1mm | NA | 19.73 | 3.52 | 17 | 21.31 | 1.81 | 10 | 1.58 | *0.138* | 2 |
| 262 | - | axial | female | wet | >1mm | NA | NA |  |  |  |  |  |  |  |  |
| 263 | <40 | axial | male | wet | >1mm | NA | NA |  |  |  |  |  |  |  |  |
|  | | | | | | |  | | | | | | | | |
| **No.** | **Age** | **Anisotropy** | **Sex** | **Condition** | **Size** | **Measuring method** | **Group 1 YM** | **SD** | **Gr 1 count (n)** | **Group 2 YM** | **SD** | **Gr 2 count (n)** | **Mean difference** | ***p*** | **Article count** |
|  | <40 vs >60 | axial vs AP vs ML | male vs female | dry vs wet | <1mm vs >1mm | bending vs strain vs compression  vs nanoindentation vs US | **Tension** |  |  | **Nanoind.** |  |  |  |  |  |
| 264 | - | - | - | - | - | NA | 15.39 | 4.24 | 87 | 21.71 | 2.49 | 105 | 6.32 | *0.000* | 13 |
| 265 | - | axial | - | - | - | NA | 17.46 | 2.89 | 57 | 20.67 | 2.45 | 47 | 3.21 | *0.000* | 9 |
| 266 | - | AP | - | - | - | NA | NA |  |  |  |  |  |  |  |  |
| 267 | - | ML | - | - | - | NA | NA |  |  |  |  |  |  |  |  |
| 268 | <40 | - | - | - | - | NA | NA |  |  |  |  |  |  |  |  |
| 269 | >60 | - | - | - | - | NA | 16.19 | 2.09 | 10 | 22.22 | 2.92 | 23 | 06.04 | *0.000* | 5 |
| 270 | - | - | male | - | - | NA | 17.44 | 4.49 | 30 | 21.71 | 2.49 | 105 | 4.27 | *0.000* | 9 |
| 271 | - | - | female | - | - | NA | NA |  |  |  |  |  |  |  |  |
| 272 | - | - | - | wet | - | NA | 16.99 | 3.73 | 50 | 20.58 | 2.40 | 46 | 3.59 | *0.000* | 8 |
| 273 | - | - | - | dry | - | NA | NA |  |  |  |  |  |  |  |  |
| 274 | - | - | - | - | <1mm | NA | NA |  |  |  |  |  |  |  |  |
| 275 | - | - | - | - | >1mm | NA | 15.39 | 4.24 | 87 | 22.59 | 2.20 | 59 | 7.20 | *0.000* | 12 |
| 276 | <40 | axial | - | - | - | NA | NA |  |  |  |  |  |  |  |  |
| 277 | >60 | axial | - | - | - | NA | NA |  |  |  |  |  |  |  |  |
| 278 | <40 | - | male | - | - | NA | NA |  |  |  |  |  |  |  |  |
| 279 | >60 | - | male | - | - | NA | NA |  |  |  |  |  |  |  |  |
| 280 | <40 | - | - | wet | - | NA | NA |  |  |  |  |  |  |  |  |
| 281 | >60 | - | - | wet | - | NA | NA |  |  |  |  |  |  |  |  |
| 282 | <40 | - | - | dry | - | NA | NA |  |  |  |  |  |  |  |  |
| 283 | >60 | - | - | dry | - | NA | NA |  |  |  |  |  |  |  |  |
| 284 | <40 | - | - | - | >1mm | NA | NA |  |  |  |  |  |  |  |  |
| 285 | >60 | - | - | - | >1mm | NA | 16.19 | 2.09 | 10 | 22.22 | 2.92 | 23 | 06.04 | *0.000* | 5 |
| 286 | - | axial | male | - | - | NA | 19.53 | 3.52 | 18 | 20.67 | 2.45 | 47 | 1.14 | *0.220* | 5 |
| 287 | - | axial | - | wet | - | NA | 17.84 | 3.25 | 38 | 20.58 | 2.40 | 46 | 2.74 | *0.000* | 6 |
| 288 | - | axial | - | dry | - | NA | NA |  |  |  |  |  |  |  |  |
| 289 | - | axial | - | - | >1mm | NA | NA |  |  |  |  |  |  |  |  |
| 290 | - | - | male | wet | - | NA | NA |  |  |  |  |  |  |  |  |
| 291 | - | - | male | dry | - | NA | NA |  |  |  |  |  |  |  |  |
| 292 | - | - | male | - | >1mm | NA | 17.44 | 4.49 | 30 | 22.59 | 2.20 | 59 | 5.15 | *0.000* | 8 |
| 293 | - | - | - | wet | >1mm | NA | NA |  |  |  |  |  |  |  |  |
| 294 | - | - | - | dry | >1mm | NA | NA |  |  |  |  |  |  |  |  |
| 295 | >60 | axial | male | - | - | NA | NA |  |  |  |  |  |  |  |  |
| 296 | >60 | axial | - | - | >1mm | NA | NA |  |  |  |  |  |  |  |  |
| 297 | <40 | - | male | dry | - | NA | NA |  |  |  |  |  |  |  |  |
| 298 | <40 | - | - | dry | >1mm | NA | NA |  |  |  |  |  |  |  |  |
| 299 | <40 | - | male | - | >1mm | NA | NA |  |  |  |  |  |  |  |  |
| 300 | - | axial | male | dry | - | NA | NA |  |  |  |  |  |  |  |  |
| 301 | - | axial | male | - | >1mm | NA | NA |  |  |  |  |  |  |  |  |
| 302 | - | axial | - | dry | >1mm | NA | NA |  |  |  |  |  |  |  |  |
| 303 | - | axial | male | wet | - | NA | 19.73 | 3.52 | 17 | 20.58 | 2.40 | 46 | 0.85 | *0.368* | 3 |
| 304 | - | axial | male | - | >1mm | NA | NA |  |  |  |  |  |  |  |  |
| 305 | - | - | male | dry | >1mm | NA | NA |  |  |  |  |  |  |  |  |
| 306 | - | axial | male | dry | >1mm | NA | NA |  |  |  |  |  |  |  |  |
| 307 | <40 |  | male | dry | >1mm | NA | NA |  |  |  |  |  |  |  |  |
| 308 | >60 |  | male | dry | >1mm | NA | NA |  |  |  |  |  |  |  |  |
|  | | | | | | |  | | | | | | | | |
| **No.** | **Age** | **Anisotropy** | **Sex** | **Condition** | **Size** | **Measuring method** | **Group 1 YM** | **SD** | **Gr 1 count (n)** | **Group 2 YM** | **SD** | **Gr 2 count (n)** | **Mean difference** | ***p*** | **Article count** |
|  | <40 vs >60 | axial vs AP vs ML | male vs female | dry vs wet | <1mm vs >1mm | bending vs strain vs compression  vs nanoindentation vs US | **Bending** |  |  | **Nanoind.** |  |  |  |  |  |
| 309 | - | - | - | - | - | NA | 12.76 | 8.85 | 49 | 21.71 | 2.49 | 105 | 8.95 | *0.000* | 8 |
| 310 | - | axial | - | - | - | NA | NA |  |  |  |  |  |  |  |  |
| 311 | - | AP | - | - | - | NA | NA |  |  |  |  |  |  |  |  |
| 312 | - | ML | - | - | - | NA | NA |  |  |  |  |  |  |  |  |
| 313 | <40 | - | - | - | - | NA | NA |  |  |  |  |  |  |  |  |
| 314 | >60 | - | - | - | - | NA | 18.80 | 3.34 | 31 | 22.22 | 2.92 | 23 | 3.42 | *0.000* | 5 |
| 315 | - | - | male | - | - | NA | 5.84 | 7.20 | 18 | 21.71 | 2.49 | 105 | 15.87 | *0.000* | 8 |
| 316 | - | - | female | - | - | NA | NA |  |  |  |  |  |  |  |  |
| 317 | - | - | - | wet | - | NA | 12.76 | 8.85 | 49 | 20.58 | 2.40 | 46 | 7.82 | *0.000* | 5 |
| 318 | - | - | - | dry | - | NA | NA |  |  |  |  |  |  |  |  |
| 319 | - | - | - | - | <1mm | NA | NA |  |  |  |  |  |  |  |  |
| 320 | - | - | - | - | >1mm | NA | 12.93 | 8.86 | 48 | 22.59 | 2.20 | 59 | 9.66 | *0.000* | 6 |
| 321 | >60 | - | male | - | - | NA | NA |  |  |  |  |  |  |  |  |
| 322 | >60 | - | - | wet | - | NA | NA |  |  |  |  |  |  |  |  |
| 323 | >60 | - | - | - | >1mm | NA | 18.80 | 3.34 | 31 | 22.22 | 2.92 | 23 | 3.42 | *0.000* | 5 |
| 324 | - | - | male | wet | - | NA | 5.84 | 7.20 | 18 | 20.58 | 2.40 | 46 | 14.74 | *0.000* | 5 |
| 325 | - | - | male | - | >1mm | NA | 5.92 | 7.41 | 17 | 22.59 | 2.20 | 59 | 16.67 | *0.000* | 6 |
| 326 | - | - | - | wet | >1mm | NA | NA |  |  |  |  |  |  |  |  |
| 327 | >60 | - | male | wet | - | NA | NA |  |  |  |  |  |  |  |  |
| 328 | >60 | - | male | - | >1mm | NA | NA |  |  |  |  |  |  |  |  |
| 329 | >60 | - | - | wet | >1mm | NA | NA |  |  |  |  |  |  |  |  |
| 330 | - | - | male | wet | >1mm | NA | NA |  |  |  |  |  |  |  |  |
| 331 | >60 | - | male | wet | >1mm | NA | NA |  |  |  |  |  |  |  |  |
|  | | | | | | |  | | | | | | | | |
| **No.** | **Age** | **Anisotropy** | **Sex** | **Condition** | **Size** | **Measuring method** | **Group 1 YM** | **SD** | **Gr 1 count (n)** | **Group 2 YM** | **SD** | **Gr 2 count (n)** | **Mean difference** | ***p*** | **Article count** |
|  | <40 vs >60 | axial vs AP vs ML | male vs female | dry vs wet | <1mm vs >1mm | bending vs strain vs compression  vs nanoindentation vs US | **Bending** |  |  | **Tension** |  |  |  |  |  |
| 332 | - | - | - | - | - | NA | 12.76 | 8.85 | 49 | 15.39 | 4.24 | 87 | 2.63 | *0.055* | 13 |
| 333 | - | axial | - | - | - | NA | NA |  |  |  |  |  |  |  |  |
| 334 | - | AP | - | - | - | NA | NA |  |  |  |  |  |  |  |  |
| 335 | - | ML | - | - | - | NA | NA |  |  |  |  |  |  |  |  |
| 336 | <40 | - | - | - | - | NA | 0.47 | 1.14 | 15 | 18.74 | 3.46 | 23 | 18.27 | *0.000* | 6 |
| 337 | >60 | - | - | - | - | NA | 18.80 | 3.34 | 31 | 16.19 | 2.09 | 10 | -2.61 | *0.007* | 4 |
| 338 | - | - | male | - | - | NA | 5.84 | 7.20 | 18 | 17.44 | 4.49 | 30 | 11.60 | *0.000* | 9 |
| 339 | - | - | female | - | - | NA | NA |  |  |  |  |  |  |  |  |
| 340 | - | - | - | wet | - | NA | 12.76 | 8.85 | 49 | 16.99 | 3.73 | 50 | 4.23 | *0.003* | 11 |
| 341 | - | - | - | dry | - | NA | NA |  |  |  |  |  |  |  |  |
| 342 | - | - | - | - | <1mm | NA | NA |  |  |  |  |  |  |  |  |
| 343 | - | - | - | - | >1mm | NA | 12.93 | 8.86 | 48 | 15.39 | 4.24 | 87 | 2.46 | *0.076* | 12 |
| 344 | <40 | AP | - | - | - | NA | NA |  |  |  |  |  |  |  |  |
| 345 | >60 | AP | - | - | - | NA | NA |  |  |  |  |  |  |  |  |
| 346 | - | AP | male | - | - | NA | NA |  |  |  |  |  |  |  |  |
| 347 | - | AP | - | wet | - | NA | NA |  |  |  |  |  |  |  |  |
| 348 | - | AP | - | - | >1mm | NA | NA |  |  |  |  |  |  |  |  |
| 349 | <40 | - | male | - | - | NA | 0.57 | 1.33 | 11 | 19.53 | 3.53 | 16 | 18.96 | *0.000* | 4 |
| 350 | <40 | - | - | wet | - | NA | 0.47 | 1.14 | 15 | 18.74 | 3.54 | 22 | 18.27 | *0.000* | 6 |
| 351 | <40 | - | - | - | >1mm | NA | 0.17 | 0.03 | 14 | 18.74 | 3.46 | 23 | 18.57 | *0.000* | 5 |
| 352 | >60 | - | male | - | - | NA | NA |  |  |  |  |  |  |  |  |
| 353 | >60 | - | - | wet | - | NA | 18.80 | 3.34 | 31 | 16.19 | 2.09 | 10 | -2.61 | *0.007* | 4 |
| 354 | >60 | - | - | - | >1mm | NA | 18.80 | 3.34 | 31 | 16.19 | 2.09 | 10 | -2.61 | *0.007* | 4 |
| 355 | - | - | male | wet | - | NA | 5.84 | 7.20 | 18 | 17.49 | 4.56 | 29 | 11.64 | *0.000* | 8 |
| 356 | - | - | male | - | >1mm | NA | 5.92 | 7.41 | 17 | 4.49 | 0.82 | 30 | 11.52 | *0.000* | 8 |
| 357 | - | - | - | wet | >1mm | NA | 12.93 | 8.86 | 48 | 16.99 | 3.73 | 50 | 04.06 | *0.005* | 10 |
| 358 | <40 | AP | - | - | >1mm | NA | NA |  |  |  |  |  |  |  |  |
| 359 | >60 | AP | - | - | >1mm | NA | NA |  |  |  |  |  |  |  |  |
| 360 | - | AP | male | - | >1mm | NA | NA |  |  |  |  |  |  |  |  |
| 361 | - | AP | - | wet | >1mm | NA | NA |  |  |  |  |  |  |  |  |
| 362 | <40 | AP | male | - | - | NA | NA |  |  |  |  |  |  |  |  |
| 363 | <40 | - | male | wet | - | NA | 0.57 | 1.33 | 11 | 19.53 | 3.53 | 16 | 18.96 | *0.000* | 4 |
| 364 | <40 | - | male | - | >1mm | NA | 0.17 | 0.03 | 10 | 19.53 | 3.53 | 16 | 19.36 | *0.000* | 3 |
| 365 | <40 | - | - | wet | >1mm | NA | 0.17 | 0.03 | 14 | 18.74 | 3.54 | 22 | 18.57 | *0.000* | 5 |
| 366 | >60 | AP | - | wet | - | NA | NA |  |  |  |  |  |  |  |  |
| 367 | >60 | - | male | wet | - | NA | NA |  |  |  |  |  |  |  |  |
| 368 | >60 | - | - | wet | >1mm | NA | 18.80 | 3.34 | 31 | 16.19 | 2.09 | 10 | -2.61 | *0.007* | 4 |
| 369 | >60 | - | male | - | >1mm | NA | NA |  |  |  |  |  |  |  |  |
| 370 | >60 | AP | - | - | >1mm | NA | NA |  |  |  |  |  |  |  |  |
| 371 | - | - | male | wet | >1mm | NA | 5.92 | 7.41 | 17 | 17.49 | 4.56 | 29 | 11.57 | *0.000* | 7 |
| 372 | - | AP | male | wet | - | NA | NA |  |  |  |  |  |  |  |  |
| 373 | <40 | AP | male | wet | - | NA | NA |  |  |  |  |  |  |  |  |
| 374 | <40 | - | male | wet | >1mm | NA | 0.17 | 0.03 | 10 | 19.53 | 3.53 | 16 | 19.36 | *0.000* | 3 |
| 375 | <40 | AP | male | - | >1mm | NA | NA |  |  |  |  |  |  |  |  |
| 376 | <40 | AP | - | wet | >1mm | NA | NA |  |  |  |  |  |  |  |  |
| 377 | >60 | AP | - | wet | >1mm | NA | NA |  |  |  |  |  |  |  |  |
| 378 | >60 | - | male | wet | >1mm | NA | NA |  |  |  |  |  |  |  |  |
| 379 | - | AP | male | wet | >1mm | NA | NA |  |  |  |  |  |  |  |  |
|  | | | | | | |  | | | | | | | | |
| **No.** | **Age** | **Anisotropy** | **Sex** | **Condition** | **Size** | **Measuring method** | **Group 1 YM** | **SD** | **Gr 1 count (n)** | **Group 2 YM** | **SD** | **Gr 2 count (n)** | **Mean difference** | ***p*** | **Article count** |
|  | <40 vs >60 | axial vs AP vs ML | male vs female | dry vs wet | <1mm vs >1mm | bending vs strain vs compression  vs nanoindentation vs US | **Bending** |  |  | **Compression** |  |  |  |  |  |
| 380 | - | - | - | - | - | NA | 12.76 | 8.85 | 49 | 17.93 | 4.14 | 31 | 5.17 | *0.001* | 8 |
| 381 | - | axial | - | - | - | NA | NA |  |  |  |  |  |  |  |  |
| 382 | - | AP | - | - | - | NA | NA |  |  |  |  |  |  |  |  |
| 383 | - | ML | - | - | - | NA | NA |  |  |  |  |  |  |  |  |
| 384 | <40 | - | - | - | - | NA | 0.47 | 1.14 | 15 | 20.09 | 2.62 | 11 | 19.62 | *0.000* | 4 |
| 385 | >60 | - | - | - | - | NA | NA |  |  |  |  |  |  |  |  |
| 386 | - | - | male | - | - | NA | 5.84 | 7.20 | 18 | 21.31 | 1.81 | 10 | 15.46 | *0.000* | 5 |
| 387 | - | - | female | - | - | NA | NA |  |  |  |  |  |  |  |  |
| 388 | - | - | - | wet | - | NA | 12.76 | 8.85 | 49 | 17.93 | 4.14 | 31 | 5.17 | *0.001* | 8 |
| 389 | - | - | - | dry | - | NA | NA |  |  |  |  |  |  |  |  |
| 390 | - | - | - | - | <1mm | NA | NA |  |  |  |  |  |  |  |  |
| 391 | - | - | - | - | >1mm | NA | 12.93 | 8.86 | 48 | 18.51 | 2.63 | 30 | 5.58 | *0.000* | 6 |
| 392 | <40 | - | male | - | - | NA | NA |  |  |  |  |  |  |  |  |
| 393 | <40 | - | - | wet | - | NA | 0.47 | 1.14 | 15 | 20.09 | 2.62 | 11 | 19.62 | *0.000* | 4 |
| 394 | <40 | - | - | - | >1mm | NA | 0.17 | 0.03 | 14 | 20.09 | 2.62 | 11 | 19.92 | *0.000* | 3 |
| 395 | - | - | male | wet | - | NA | 5.84 | 7.20 | 18 | 21.31 | 1.81 | 10 | 15.46 | *0.000* | 5 |
| 396 | - | - | male | - | >1mm | NA | 5.92 | 7.41 | 17 | 21.31 | 1.81 | 10 | 15.39 | *0.000* | 4 |
| 397 | - | - | - | wet | >1mm | NA | 12.93 | 8.86 | 48 | 18.51 | 2.63 | 30 | 5.58 | *0.000* | 6 |
| 398 | <40 | - | - | wet | >1mm | NA | 0.17 | 0.03 | 14 | 20.09 | 2.62 | 11 | 19.92 | *0.000* | 3 |
| 399 | - | - | male | wet | >1mm | NA | 5.92 | 7.41 | 17 | 21.31 | 1.81 | 10 | 15.39 | *0.000* | 4 |
|  | | | | | | |  | | | | | | | | |
| **No.** | **Age** | **Anisotropy** | **Sex** | **Condition** | **Size** | **Measuring method** | **Group 1 YM** | **SD** | **Gr 1 count (n)** | **Group 2 YM** | **SD** | **Gr 2 count (n)** | **Mean difference** | ***p*** | **Article count** |
|  | <40 vs >60 | axial vs AP vs ML | male vs female | dry vs wet | <1mm vs >1mm | bending vs strain vs compression  vs nanoindentation vs US | **Nanoind.** |  |  | **Compression** |  |  |  |  |  |
| 400 | - | - | - | - | - | NA | 21.71 | 2.49 | 105 | 17.93 | 4.14 | 31 | -3.78 | *0.000* | 8 |
| 401 | - | axial | - | - | - | NA | 20.67 | 2.45 | 47 | 18.51 | 2.63 | 30 | -2.16 | *0.001* | 5 |
| 402 | - | AP | - | - | - | NA | NA |  |  |  |  |  |  |  |  |
| 403 | - | ML | - | - | - | NA | NA |  |  |  |  |  |  |  |  |
| 404 | <40 | - | - | - | - | NA | NA |  |  |  |  |  |  |  |  |
| 405 | >60 | - | - | - | - | NA | NA |  |  |  |  |  |  |  |  |
| 406 | - | - | male | - | - | NA | 21.71 | 2.49 | 105 | 21.31 | 1.81 | 10 | -0.40 | *0.527* | 5 |
| 407 | - | - | female | - | - | NA | NA |  |  |  |  |  |  |  |  |
| 408 | - | - | - | wet | - | NA | 20.58 | 2.40 | 46 | 17.93 | 4.14 | 31 | -2.65 | *0.002* | 5 |
| 409 | - | - | - | dry | - | NA | NA |  |  |  |  |  |  |  |  |
| 410 | - | - | - | - | <1mm | NA | NA |  |  |  |  |  |  |  |  |
| 411 | - | - | - | - | >1mm | NA | NA |  |  |  |  |  |  |  |  |
| 412 | - | axial | male | wet | - | NA | 20.58 | 2.40 | 46 | 21.31 | 1.81 | 10 | 0.73 | *0.296* | 2 |
|  |  |  |  |  |  |  |  |  |  |  |  |  |  |  |  |
| **Is there a difference between bone ends in knee joint ?** | | | | | | | | | | | | | | | |
| **Distal femur vs proximal tibia, epiphysis, cancellous** | | | | | | |  |  |  |  |  |  |  |  |  |
| **No.** | **Age** | **Anisotropy** | **Sex** | **Condition** | **Size** | **Measuring method** | **Group 1 YM** | **SD** | **Gr 1 count (n)** | **Group 2 YM** | **SD** | **Gr 2 count (n)** | **Mean difference** | ***p*** | **Article count** |
|  | <40 vs >60 | axial vs AP vs ML | male vs female | dry vs wet | <1mm vs >1mm | bending vs strain vs compression  vs nanoindentation vs US | **Distal epiphysis of femur** |  |  | **Proximal epiphysis of tibia** |  |  |  |  |  |
| 413 | - | - | - | - | - | - | 0.39 | 0.40 | 39 | 01.06 | 1.21 | 20 | 0.67 | *0.026* | 3 |
| 414 | <40 | - | - | - | - | - | NA |  |  |  |  |  |  |  |  |
| 415 | >60 | - | - | - | - | - | NA |  |  |  |  |  |  |  |  |
| 416 | - | axial | - | - | - | - | 0.39 | 0.40 | 39 | 0.69 | 0.44 | 18 | 0.30 | *0.019* | 2 |
| 417 | - | AP | - | - | - | - | NA |  |  |  |  |  |  |  |  |
| 418 | - | ML | - | - | - | - | NA |  |  |  |  |  |  |  |  |
| 419 | - | - | male | - | - | - | 0.39 | 0.40 | 39 | 01.06 | 1.21 | 20 | 0.67 | *0.026* | 3 |
| 420 | - | - | female | - | - | - | NA |  |  |  |  |  |  |  |  |
| 421 | - | - | - | wet | - | - | 0.39 | 0.40 | 39 | 01.06 | 1.21 | 20 | 0.67 | *0.026* | 3 |
| 422 | - | - | - | dry | - | - | NA |  |  |  |  |  |  |  |  |
| 423 | - | - | - | - | <1mm | - | NA |  |  |  |  |  |  |  |  |
| 424 | - | - | - | - | >1mm | - | 0.39 | 0.40 | 39 | 0.69 | 0.44 | 18 | 0.30 | *0.019* | 2 |
| 425 | - | - | - | - | - | bending | NA |  |  |  |  |  |  |  |  |
| 426 | - | - | - | - | - | tension | NA |  |  |  |  |  |  |  |  |
| 427 | - | - | - | - | - | compression | 0.39 | 0.40 | 39 | 0.69 | 0.44 | 18 | 0.30 | *0.019* | 2 |
| 428 | - | - | - | - | - | nanoidentitation | NA |  |  |  |  |  |  |  |  |
| 429 | - | - | - | - | - | ultrasound | NA |  |  |  |  |  |  |  |  |
| 430 | - | axial | male | wet | >1mm | compression | 0.39 | 0.40 | 39 | 0.69 | 0.44 | 18 | 0.30 | *0.019* | 2 |
|  | | | | | | |  | | | | | | | | |
| **Distal femur vs proximal tibia, epiphysis, cancellous** | | | | | | |  | | | | | | | | |
| **No.** | **Age** | **Anisotropy** | **Sex** | **Condition** | **Size** | **Measuring method** | **Group 1 YM** | **SD** | **Gr 1 count (n)** | **Group 2 YM** | **SD** | **Gr 2 count (n)** | **Mean difference** | ***p*** | **Article count** |
|  | <40 vs >60 | axial vs AP vs ML | male vs female | dry vs wet | <1mm vs >1mm | bending vs strain vs compression  vs nanoindentation vs US | **Distal epiphysis of femur** |  |  | **Proximal epiphysis of tibia** |  |  |  |  |  |
| 431 | - | - | - | - | - | - | NA |  |  |  |  |  |  |  |  |
|  |  |  |  |  |  |  |  |  |  |  |  |  |  |  |  |
| **Is there a difference between same bone, different regions ?** | | | | | | | | | | | | | | | |
| **Femur epiphysis vs metaphysis vs diaphysis, cortical** | | | | | | |  | | | | | | | | |
| **No.** | **Age** | **Anisotropy** | **Sex** | **Condition** | **Size** | **Measuring method** | **Group 1 YM** | **SD** | **Gr 1 count (n)** | **Group 2 YM** | **SD** | **Gr 2 count (n)** | **Mean difference** | ***p*** | **Article count** |
|  | <40 vs >60 | axial vs AP vs ML | male vs female | dry vs wet | <1mm vs >1mm | bending vs strain vs compression  vs nanoindentation vs US | **epiphysis** |  |  | **metaphysis** |  |  |  |  |  |
| 432 | - | - | - | - | - | - | NA |  |  |  |  |  |  |  |  |
|  |  |  |  |  |  |  | **epiphysis** |  |  | **diaphysis** |  |  |  |  |  |
| 433 | - | - | - | - | - | - | NA |  |  |  |  |  |  |  |  |
|  |  |  |  |  |  |  | **metaphysis** |  |  | **diaphysis** |  |  |  |  |  |
| 434 | - | - | - | - | - | - | NA |  |  |  |  |  |  |  |  |
|  | | | | | | |  | | | | | | | | |
| **Femur epiphysis vs metaphysis vs diaphysis, cancellous** | | | | | | |  | | | | | | | | |
| **No.** | **Age** | **Anisotropy** | **Sex** | **Condition** | **Size** | **Measuring method** | **Group 1 YM** | **SD** | **Gr 1 count (n)** | **Group 2 YM** | **SD** | **Gr 2 count (n)** | **Mean difference** | ***p*** | **Article count** |
|  | <40 vs >60 | axial vs AP vs ML | male vs female | dry vs wet | <1mm vs >1mm | bending vs strain vs compression  vs nanoindentation vs US | **epiphysis** |  |  | **diaphysis** |  |  |  |  |  |
| 435 | - | - | - | - | - | - | NA |  |  |  |  |  |  |  |  |
|  |  |  |  |  |  |  | **metaphysis** |  |  | **diaphysis** |  |  |  |  |  |
| 436 | - | - | - | - | - | - | NA |  |  |  |  |  |  |  |  |
|  |  |  |  |  |  |  | **epiphysis** |  |  | **metaphysis** |  |  |  |  |  |
| 437 | - | - | - | - | - | - | 0.87 | 2.35 | 97 | 3.01 | 5.05 | 44 | 2.13 | *0.010* | 9 |
| 438 | <40 | - | - | - | - | - | NA |  |  |  |  |  |  |  |  |
| 439 | >60 | - | - | - | - | - | 1.24 | 3.27 | 48 | 4.00 | 5.84 | 21 | 2.77 | *0.052* | 6 |
| 440 | - | axial | - | - | - | - | 0.92 | 3.25 | 47 | 0.25 | 0.22 | 14 | -0.68 | *0.163* | 3 |
| 441 | - | AP | - | - | - | - | NA |  |  |  |  |  |  |  |  |
| 442 | - | ML | - | - | - | - | NA |  |  |  |  |  |  |  |  |
| 443 | - | - | male | - | - | - | 0.63 | 0.50 | 65 | 2.20 | 4.58 | 24 | 1.56 | *0.109* | 4 |
| 444 | - | - | female | - | - | - | 3.38 | 6.56 | 11 | 4.59 | 6.04 | 16 | 1.20 | *0.634* | 6 |
| 445 | - | - | - | wet | - | - | 0.75 | 0.83 | 75 | 2.63 | 4.81 | 38 | 1.88 | *0.022* | 5 |
| 446 | - | - | - | dry | - | - | NA |  |  |  |  |  |  |  |  |
| 447 | - | - | - | - | <1mm | - | NA |  |  |  |  |  |  |  |  |
| 448 | - | - | - | - | >1mm | - | 0.87 | 2.35 | 97 | 0.30 | 0.38 | 30 | -0.58 | *0.021* | 6 |
| 449 | - | - | - | - | - | bending | NA |  |  |  |  |  |  |  |  |
| 450 | - | - | - | - | - | tension | NA |  |  |  |  |  |  |  |  |
| 451 | - | - | - | - | - | compression | 0.59 | 0.49 | 95 | 0.40 | 0.50 | 33 | -0.19 | *0.068* | 5 |
| 452 | - | - | - | - | - | nanoidentitation | NA |  |  |  |  |  |  |  |  |
| 453 | - | - | - | - | - | ultrasound | NA |  |  |  |  |  |  |  |  |
| 454 | - | - | - | wet | >1mm | - | 0.75 | 0.83 | 75 | 0.30 | 0.38 | 30 | -0.45 | *0.000* | 4 |
| 455 | - | - | - | wet | - | compression | 0.67 | 0.52 | 74 | 0.30 | 0.38 | 30 | -0.37 | *0.000* | 3 |
| 456 | - | - | - | - | >1mm | compression | 0.59 | 0.49 | 95 | 0.30 | 0.38 | 30 | -0.29 | *0.001* | 4 |
| 457 | - | - | - | wet | >1mm | compression | 0.67 | 0.52 | 74 | 0.30 | 0.38 | 30 | -0.37 | *0.000* | 3 |

**Table_S3**. Risk of bias assessment using the CARE tool (Gagnier, J.J., et al., *The CARE Guidelines: Consensus-based Clinical Case Reporting Guideline Development.* Glob Adv Health Med, 2013. **2**(5): p. 38-43.)

|  | Study (year) | Domain 1 | Domain 2 | Domain 3 | Domain 4 | Domain 5 | Domain 6 | Domain 7 | Domain 8 |
| --- | --- | --- | --- | --- | --- | --- | --- | --- | --- |
| 1 | Anglin (1999) |  |  |  |  |  |  |  |  |
| 2 | Ashman (1988) |  |  |  |  |  |  |  |  |
| 3 | Banse (1996) |  |  |  |  |  |  |  |  |
| 4 | Bargren (1974) |  |  |  |  |  |  |  |  |
| 5 | Bensamoun (2008) |  |  |  |  |  |  |  |  |
| 6 | Bensamoun (2004) |  |  |  |  |  |  |  |  |
| 7 | Bini 2002 |  |  |  |  |  |  |  |  |
| 8 | Birnbaum (2001) |  |  |  |  |  |  |  |  |
| 9 | Bry (2012) |  |  |  |  |  |  |  |  |
| 10 | Carretta (2013) |  |  |  |  |  |  |  |  |
| 11 | Choi (1990) |  |  |  |  |  |  |  |  |
| 12 | Cuppone (2004) |  |  |  |  |  |  |  |  |
| 13 | Dall'Ara (2013) |  |  |  |  |  |  |  |  |
| 14 | Dong (2004) |  |  |  |  |  |  |  |  |
| 15 | Ducheyne 1977) |  |  |  |  |  |  |  |  |
| 16 | Dunham 2005) |  |  |  |  |  |  |  |  |
| 17 | Evans 1976) |  |  |  |  |  |  |  |  |
| 18 | Fan 2002) |  |  |  |  |  |  |  |  |
| 19 | Fan 2003) |  |  |  |  |  |  |  |  |
| 20 | Franzoso (2009) |  |  |  |  |  |  |  |  |
| 21 | Guérard (2011) |  |  |  |  |  |  |  |  |
| 22 | Hengsberger (2001) |  |  |  |  |  |  |  |  |
| 23 | Jensen (1988) |  |  |  |  |  |  |  |  |
| 24 | Katsamanis (1990) |  |  |  |  |  |  |  |  |
| 25 | Keller (1990) |  |  |  |  |  |  |  |  |
| 26 | Korsa (2015) |  |  |  |  |  |  |  |  |
| 27 | Kuhn (1989) |  |  |  |  |  |  |  |  |
| 28 | Mansat (1998) |  |  |  |  |  |  |  |  |
| 29 | Martens (1983) |  |  |  |  |  |  |  |  |
| 30 | Moreschi (2011) |  |  |  |  |  |  |  |  |
| 31 | Nomura (2007) |  |  |  |  |  |  |  |  |
| 32 | Odgaard (1989) |  |  |  |  |  |  |  |  |
| 33 | Odgaard (1991) |  |  |  |  |  |  |  |  |
| 34 | Pattijn (2001) |  |  |  |  |  |  |  |  |
| 35 | Pattin (1996) |  |  |  |  |  |  |  |  |
| 36 | Reilly (1974) |  |  |  |  |  |  |  |  |
| 37 | Reilly (1975) |  |  |  |  |  |  |  |  |
| 38 | Reisinger (2020) |  |  |  |  |  |  |  |  |
| 39 | Rho (1997) |  |  |  |  |  |  |  |  |
| 40 | Rho (1999) |  |  |  |  |  |  |  |  |
| 41 | Rho (2002) |  |  |  |  |  |  |  |  |
| 42 | Rho (1993) |  |  |  |  |  |  |  |  |
| 43 | Sedlin (1966) |  |  |  |  |  |  |  |  |
| 44 | Stern (2011) |  |  |  |  |  |  |  |  |
| 45 | Subit (2013) |  |  |  |  |  |  |  |  |
| 46 | Winwood (2006) |  |  |  |  |  |  |  |  |
| 47 | Zysset (1998) |  |  |  |  |  |  |  |  |

**Domains description:
Domain 1 - Were there clear criteria for inclusion in the case series?
Domain 2 - Was the condition measured in a standard, reliable way for all participants included in the case series?
Domain 3 - Were valid methods used for identification of the condition for all participants included in the case series?
Domain 4 - Did the case series have consecutive "következetes" inclusion of participants?
Domain 5 - Did the case series have complete inclusion of participants?
Domain 6 - Was there clear reporting of the demographics of the participants in the study?
Domain 7 - Were the outcomes or follow up results of cases clearly reported?
Domain 8 - Was there clear reporting of the presenting site(s)/clinic(s) demographic information?**

**The CARE tool contains two more domains, however, those are not applicable to our study.**

(Was there clear reporting of clinical information of the participants?; Was statistical analysis appropriate?),

**Table_S4.** Supplementary table of unfavorable data structure example in case of femur diaphysis. In each cell, the entry count numbers are represented.

**REFERENCES**

1. Anglin C, Tolhurst P, Wyss UP, Pichora DR. Glenoid cancellous bone strength and modulus. *Journal of biomechanics.* 1999;32(10):1091-1097.

2. Ashman RB, Jae Young R. Elastic modulus of trabecular bone material. *Journal of biomechanics.* 1988;21(3):177-181.

3. Banse X, Delloye C, Cornu O, Bourgois R. Comparative left-right mechanical testing of cancellous bone from normal femoral heads. *Journal of biomechanics.* 1996;29(10):1247-1253.

4. Bargren JH, Bassett CAL, Gjelsvik A. Mechanical properties of hydrated cortical bone. *Journal of biomechanics.* 1974;7(3):239-245.

5. Bensamoun S, Fan Z, Brice I, Rho JY, Tho M-CHB. ASSESSMENT OF MECHANICAL PROPERTIES OF HUMAN OSTEON LAMELLAE EXHIBITING VARIOUS DEGREES OF MINERALIZATION BY NANOINDENTATION. 2008;11(03):135-143.

6. Bensamoun S, Ho Ba Tho M-C, Luu S, Gherbezza J-M, de Belleval J-F. Spatial distribution of acoustic and elastic properties of human femoral cortical bone. *Journal of biomechanics.* 2004;37(4):503-510.

7. Berteau J-P, Baron C, Pithioux M, Launay F, Chabrand P, Lasaygues P. In vitro ultrasonic and mechanic characterization of the modulus of elasticity of children cortical bone. *Ultrasonics.* 2014;54(5):1270-1276.

8. Bini F, Marinozzi A, Marinozzi F, Patanè F. Microtensile measurements of single trabeculae stiffness in human femur. *Journal of biomechanics.* 2002;35(11):1515-1519.

9. Birnbaum K, Sindelar R, Gaertner JR, Wirtz DC. Material properties of trabecular bone structures. *Surgical and radiologic anatomy : SRA.* 2001;23(6):399-407.

10. Bry R, Bennani B, Delille R, Morvan H, Hault-Dubrulle A, Fontaine C. Mechanical characterisation under cycling loading of humerus cortical bone. *Computer Methods in Biomechanics and Biomedical Engineering.* 2012;15(sup1):274-276.

11. Carretta R, Stüssi E, Müller R, Lorenzetti S. Within subject heterogeneity in tissue-level post-yield mechanical and material properties in human trabecular bone. *Journal of the Mechanical Behavior of Biomedical Materials.* 2013;24:64-73.

12. Choi K, Kuhn JL, Ciarelli MJ, Goldstein SA. The elastic moduli of human subchondral, trabecular, and cortical bone tissue and the size-dependency of cortical bone modulus. *Journal of biomechanics.* 1990;23(11):1103-1113.

13. Cuppone M, Seedhom BB, Berry E, Ostell AE. The Longitudinal Young’s Modulus of Cortical Bone in the Midshaft of Human Femur and its Correlation with CT Scanning Data. *Calcified Tissue International.* 2004;74(3):302-309.

14. Dall'Ara E, Karl C, Mazza G, et al. Tissue properties of the human vertebral body sub-structures evaluated by means of microindentation. *Journal of the Mechanical Behavior of Biomedical Materials.* 2013;25:23-32.

15. Ducheyne P, Heymans L, Martens M, Aernoudt E, de Meester P, Mulier JC. The mechanical behaviour of intracondylar cancellous bone of the femur at different loading rates. *Journal of biomechanics.* 1977;10(11):747-762.

16. Dunham CE, Takaki SE, Johnson JA, Dunning CE. Mechanical properties of cancellous bone of the distal humerus. *Clinical Biomechanics.* 2005;20(8):834-838.

17. Evans FG. Mechanical properties and histology of cortical bone from younger and older men. *The Anatomical Record.* 1976;185(1):1-11.

18. Fan Z, Rho J-Y. Effects of viscoelasticity and time-dependent plasticity on nanoindentation measurements of human cortical bone. *Journal of Biomedical Materials Research Part A.* 2003;67A(1):208-214.

19. Fan Z, Swadener JG, Rho JY, Roy ME, Pharr GM. Anisotropic properties of human tibial cortical bone as measured by nanoindentation. 2002;20(4):806-810.

20. Franzoso G, Zysset PK. Elastic anisotropy of human cortical bone secondary osteons measured by nanoindentation. *Journal of biomechanical engineering.* 2009;131(2):021001.

21. Guérard S, Chevalier Y, Moreschi H, Defontaine M, Callé S, Mitton D. Young's modulus repeatability assessment using cycling compression loading on cancellous bone. *Proceedings of the Institution of Mechanical Engineers Part H, Journal of engineering in medicine.* 2011;225(11):1113-1117.

22. Hengsberger S, Kulik A, Zysset P. A combined atomic force microscopy and nanoindentation technique to investigate the elastic properties of bone structural units. *European cells & materials.* 2001;1:12-17.

23. Jensen NC, Hvid I, Krøner K. Strength pattern of cancellous bone at the ankle joint. *Engineering in medicine.* 1988;17(2):71-76.

24. Katsamanis F, Raftopoulos DD. Determination of mechanical properties of human femoral cortical bone by the Hopkinson bar stress technique. *Journal of biomechanics.* 1990;23(11):1173-1184.

25. Keller TS, Mao Z, Spengler DM. Young's modulus, bending strength, and tissue physical properties of human compact bone. *Journal of Orthopaedic Research.* 1990;8(4):592-603.

26. Korsa R, Lukes J, Sepitka J, Mares T. Elastic Properties of Human Osteon and Osteonal Lamella Computed by a Bidirectional Micromechanical Model and Validated by Nanoindentation. *Journal of biomechanical engineering.* 2015;137(8):081002.

27. Kuhn JL, Goldstein SA, Choi R, London M, Feldkamp LA, Matthews LS. Comparison of the trabecular and cortical tissue moduli from human iliac crests. *Journal of Orthopaedic Research.* 1989;7(6):876-884.

28. Mansat P, Barea C, Hobatho M-C, Darmana R, Mansat M. Anatomic variation of the mechanical properties of the glenoid. *Journal of Shoulder and Elbow Surgery.* 1998;7(2):109-115.

29. Martens M, Van Audekercke R, Delport P, De Meester P, Mulier JC. The mechanical characteristics of cancellous bone at the upper femoral region. *Journal of biomechanics.* 1983;16(12):971-983.

30. Moreschi H, Callé S, Guerard S, Mitton D, Renaud G, Defontaine M. Monitoring Trabecular Bone Microdamage Using a Dynamic Acousto-Elastic Testing Method. *Proceedings of the Institution of Mechanical Engineers, Part H: Journal of Engineering in Medicine.* 2010;225(3):282-295.

31. Neil Dong X, Edward Guo X. The dependence of transversely isotropic elasticity of human femoral cortical bone on porosity. *Journal of biomechanics.* 2004;37(8):1281-1287.

32. Nomura T, Katz JL, Powers MP, Saito C. A micromechanical elastic property study of trabecular bone in the human mandible. *Journal of Materials Science: Materials in Medicine.* 2007;18(4):629-633.

33. Odgaard A, Hvid I, Linde F. Compressive axial strain distributions in cancellous bone specimens. *Journal of biomechanics.* 1989;22(8):829-835.

34. Odgaard A, Linde F. The underestimation of Young's modulus in compressive testing of cancellous bone specimens. *Journal of biomechanics.* 1991;24(8):691-698.

35. Pattijn V, Van Cleynenbreugel T, Sloten JV, Van Audekercke R, Van der Perre G, Wevers M. Structural and Radiological Parameters for the Nondestructive Characterization of Trabecular Bone. *Annals of biomedical engineering.* 2001;29(12):1064-1073.

36. Pattin CA, Caler WE, Carter DR. Cyclic mechanical property degradation during fatigue loading of cortical bone. *Journal of biomechanics.* 1996;29(1):69-79.

37. Reilly DT, Burstein AH. The elastic and ultimate properties of compact bone tissue. *Journal of biomechanics.* 1975;8(6):393-405.

38. Reilly DT, Burstein AH, Frankel VH. The elastic modulus for bone. *Journal of biomechanics.* 1974;7(3):271-275.

39. Reisinger AG, Frank M, Thurner PJ, Pahr DH. A two-layer elasto-visco-plastic rheological model for the material parameter identification of bone tissue. *Biomechanics and Modeling in Mechanobiology.* 2020;19(6):2149-2162.

40. Rho JY, Ashman RB, Turner CH. Young's modulus of trabecular and cortical bone material: Ultrasonic and microtensile measurements. *Journal of biomechanics.* 1993;26(2):111-119.

41. Rho JY, Zioupos P, Currey JD, Pharr GM. Variations in the individual thick lamellar properties within osteons by nanoindentation. *Bone.* 1999;25(3):295-300.

42. Rho JY, Zioupos P, Currey JD, Pharr GM. Microstructural elasticity and regional heterogeneity in human femoral bone of various ages examined by nano-indentation. *Journal of biomechanics.* 2002;35(2):189-198.

43. Rho J-Y, Tsui TY, Pharr GM. Elastic properties of human cortical and trabecular lamellar bone measured by nanoindentation. *Biomaterials.* 1997;18(20):1325-1330.

44. Sedlin ED, Hirsch C. Factors Affecting the Determination of the Physical Properties of Femoral Cortical Bone. *Acta Orthopaedica Scandinavica.* 1966;37(1):29-48.

45. Stern LC, Brinkman JG, Furmanski J, Rimnac CM, Hernandez CJ. Near-terminal creep damage does not substantially influence fatigue life under physiological loading. *Journal of biomechanics.* 2011;44(10):1995-1998.

46. Subit D, Dios E, Velazquez-Ameijide J, Arregui-Dalmases C, Crandall J. Tensile material properties of human rib cortical bone under quasi-static and dynamic failure loading and influence of the bone microstucture on failure characteristics. 2011.

47. Winwood K, Zioupos P, Currey JD, Cotton JR, Taylor M. Strain patterns during tensile, compressive, and shear fatigue of human cortical bone and implications for bone biomechanics. *Journal of Biomedical Materials Research Part A.* 2006;79A(2):289-297.

48. Zysset P, Guo XE, Hoffler C, Moore K, Goldstein S. Mechanical properties of human trabecular bone lamellae quantified by nanoindentation. *Technology and health care : official journal of the European Society for Engineering and Medicine.* 1999;6:429-432.
